# Distinct characteristics of VEXAS-causative *UBA1* M41 and recurrent functional non-M41 mutations

**DOI:** 10.1038/s41375-025-02775-4

**Published:** 2025-10-09

**Authors:** Maki Sakuma, Amy K. Wang, Samuel J. Magaziner, Sachiko P. Keane, Manja Meggendorfer, Wolfgang Kern, Claudia Haferlach, Torsten Haferlach, David B. Beck, Wencke Walter

**Affiliations:** 1 Munich Leukemia Laboratory, Munich, Germany; 2 Graduate School of Medicine and Health, Technical University of Munich, Munich, Germany; 3 Center for Human Genetics and Genomics, NYU School of Medicine, New York, NY USA; 4 Department of Medicine, NYU School of Medicine, New York, NY USA; 5 Department of Biochemistry and Molecular Pharmacology, NYU School of Medicine, New York, NY USA

**Keywords:** Leukaemia, Haematopoiesis

## Abstract

VEXAS (vacuoles, E1 enzyme, X-linked, autoinflammatory, somatic) syndrome is a severe inflammatory and hematologic disease caused by somatic mutations in *UBA1*. Canonical pathogenic mutations at *UBA1* p.Met41 (M41) lead to the loss of the cytoplasmic isoform (UBA1b), while non-canonical mutations outside of M41 (non-M41) result in reduced activity of both nuclear and cytoplasmic isoforms. Studies have reported clinical differences between canonical and non-canonical mutations, but these findings are constrained by small sample sizes and scarcity of genetic studies. In our study, we screened 29,000 individuals for *UBA1* variants, referred for a broad range of hematologic diseases, and subjected to 62-gene panel sequencing, identifying 232 patients carrying likely disease-causing mutations. We identified decreased polyubiquitylation in all of the 18 *UBA1* variants tested and found differences in H2A/B monoubiquitylation alteration between M41 and non-M41 mutations. Our findings confirm that patients harboring M41 mutations present at most with myelodysplastic neoplasms (MDS) and suggest that M41 mutations generally do not tolerate multiple co-mutations. In contrast, non-M41 mutations are more likely to appear with co-mutations and are detected in patients with hematologic neoplasms other than MDS. Our study establishes that M41 and non-M41 mutations exhibit distinct clinical and biological phenotypes, significantly enhancing *UBA1* variant interpretation.

## Introduction

Identified in late 2020, VEXAS (vacuoles, E1 enzyme, X-linked, autoinflammatory, somatic) syndrome is a hemato-inflammatory disorder caused by hypomorphic mutations in *UBA1*, a crucial E1 enzyme within the ubiquitin-proteasome system [1]. This condition poses significant clinical challenges due to treatment refractory severe inflammation and progressive clonal cytopenia [2], even though VEXAS is considered low-risk of AML transformation.

*UBA1* disease-causing mutations are categorized into two distinct classes: mutations near p.Met41 (M41), affecting the translational start codon of the cytoplasmic isoform (UBA1b) and leading to an exclusive loss of cytoplasmic activity, and non-M41 mutations, which result in varying degrees of enzymatic dysfunction for both nuclear and cytoplasmic isoforms [3]. The M41 mutations consist of M41V, M41L, M41T, and the neighboring splice site variants that alter translation of UBA1b [4]. While M41 mutations are unanimously recognized as causative of VEXAS, the role of non-M41 mutations remains contentious due to the lack of diagnostic criteria and limited numbers of patients detected in previous case series [5, 6] and cohorts [7–10]. Additionally, it remains unclear whether there are clinical differences in inflammation, risk of hematologic malignancies (HM), and severity of cytopenias between mutation types. Furthermore, as comprehensive sequencing of *UBA1* (entire coding sequence) becomes more common [11], the detection of additional uncharacterized non-M41 variants poses diagnostic challenges.

This study aims to provide a detailed comparison of 151 and 81 patients harboring M41 and non-M41 mutations, respectively, regarding their clinical manifestations and biochemical consequences. We provide functional evidence of 18 *UBA1* non-M41 variants and find differences in H2A/B monoubiquitylation between the two classes of variants. We confirm the low-risk of AML conversion for M41 mutations and find that they generally do not occur with multiple co-dominant mutations. Conversely, the non-M41 mutations showed higher frequency of HM and co-mutations. In isolation, non-M41 mutations showed increased erythropoiesis and more severe thrombocytopenia compared to M41 mutations. Our findings suggest different mechanisms of action for *UBA1* mutations, with distinct phenotypes, indicating distinct pathophysiological modes.

## Materials and methods

### Patient cohort

The cohort consisted of 164,320 patients received at the MLL Munich Leukemia Laboratory for diagnostic testing between July 1st 2022 and 30th September 2024. The patients were divided into 135,320 patients without targeted myeloid gene panel analysis and 29,000 patients with, further subdivided into 17,714 initial diagnosis (ID), 11,286 follow-up only (FU). When the first sample received for ID was inconclusive (e.g. due to insufficient material), the following treatment-native samples received within 100 days were considered ID samples. When multiple samples were sequenced for *UBA1* from the same individual, the results from the bone marrow (BM) were prioritized (N = 7). Patients additionally underwent standard cytomorphological, cytogenetic, immunophenotyping testing, performed as described in Baumgartner et al. [12] based on requests. The data availability and sample characteristics are given in Supplementary Table 1.

### Diagnosis

Patients were diagnosed following WHO 2022 Classification [13]. When the samples underwent mutational or cytogenetic analyses only, the diagnosis was “unassigned” due to lack of information, unless genetic information suffices for the diagnosis. Pre/non-HM included benign entities, monoclonal B lymphocytosis (MBL), monoclonal gammopathy of undetermined significance (MGUS), clonal cytopenia of undetermined significance (CCUS) and other morphologically non-diagnostic cases.

### Sequencing

Targeted myeloid gene panel sequencing consisted of 62 genes given in Supplementary Table 2. All *UBA1*-positive samples underwent panel sequencing at first sample reception. From the second time on, samples with known exon 3 variants underwent amplicon sequencing for *UBA1* exon 3 along with specific co-mutations detected (M41 N = 8, S56F N = 1), unless the panel sequencing was requested (M41 N = 9, S56F N = 1). Non-exon 3 variants underwent panel sequencing. Amplicon sequencing was performed on MiSeq platform and variants were called with SeqNext 4.3 (JSI Medical Systems, Kippenheim, Germany). Panel sequencing was performed on NovaSeq6000 after Illumina DNA library preparation (Illumina, San Diego, CA) and enrichment with a custom panel by IDT (Integrated DNA Technologies, Iowa, USA) following manufacturer’s instructions. Gene variants were called with Pisces (available via BaseSpace, Illumina), using a sensitivity level of 3%. In known pathogenic variants with VAF < 3%, a second sequencing was performed, and samples with twice VAF > 1% were called positive.

### Variant analysis

For variant annotation several databases (in-house, COSMIC [14], IARC [15] and ClinVAR [16]) and in silico predictions were used. Pathogenicity of mutations was assessed based on a 4-tier system, with Tier-1 being clearly pathogenic (P), Tier-2 likely pathogenic (LP), Tier-3 variants of unknown significance (VUS), and Tier-4 polymorphisms (gnomAD [17] frequency >0.05%) and synonymous variants. The number of co-mutations is the sum of the co-occurring variants classified as Tier-1 or Tier-2 for all 62 genes in the myeloid gene panel.

All *UBA1* M41-affecting variants were classified as M41 mutations. *UBA1* non-M41 variants on recurrently somatic Tier-1, Tier-2, or Tier-3 loci were compared against M41, where somatic variants were defined as VAF < 95% for males and in addition outside of VAF between 35% and 55% for females. Chimeric genetic constitution due to allogenic stem cell transplantation were excluded. Frequency of recurrent variants, *UBA1* variant classification and patient information is given in Supplementary Table 3 and 4.

To compare with *UBA1* variants, specific established variants associated with clonal hematopoiesis of indeterminate potential (CHIP) and myelodysplastic neoplasms (MDS) were selected from the literature as follows: *DNMT3A* R882/LOF, *TET2* LOF, *ASXL1* LOF, *SF3B1* K700/K666, *SRSF2* P95, *ZRSR2* LOF, *U2AF1* S34/Q157, *BCOR* LOF, *EZH2* LOF, *STAG2* LOF, *TP53* R273/LOF [18, 19]. Loss of function (LOF) variants are defined as nonsense, frameshift, and canonical splice variants. VAF was adjusted to half for males for genes on the X chromosome as well as VAF > 50%. The VAF of patients carrying multiple variants in the same gene was represented by the highest VAF among the pathogenic variants.

### Co-mutation analysis

For patients harboring multiple co-mutations, the co-mutation with the highest VAF was selected. Patients were separated into the following four groups: those harboring *DNMT3A* mutations, *TET2* mutations, MDS-associated mutations (*ASXL1*, *BCOR*, *EZH2*, *STAG2*, *SF3B1*, *SRSF2*, *ZRSR2*, *U2AF1*), and other mutations. For each group, the patients were classified as co-dominant (both VAF ≥ 15%), *UBA1*-dominant (*UBA1* VAF ≥ 15%, co-mutation VAF < 15%), co-mutation dominant (*UBA1* VAF < 15%, co-mutation VAF ≥ 15%), and non-dominant (both VAF < 15%).

### Bone marrow and blood count analysis

The reference group was created by taking patients who underwent genetic and morphologic analysis of BM samples and for whom no mutations or chromosomal aberrations were detected (N = 1598). For the blood count analysis, patients were restricted to those with WBC, Hb, and PLT available (N = 1466), and the proportion of patients satisfying specific criteria was compared to the reference by Fisher’s test with Benjamin-Hochberg correction. For *UBA1*-mutated patients, difference in individual non-normalized values of the parameters was tested by Wilcoxon rank sum test with Benjamin-Hochberg correction. All statistical tests were performed in R.

### Cell lines and media

Parental Chinese hamster ovary (CHO) cell line (e36) and temperature sensitive UBA1 knockdown CHO cell line (ts20) [20] were cultured in complete CHO ts20 medium (MEM α; Gibco, 12571063 supplemented with 1.8 g/L glucose, 10% FBS, and Penicillin-Streptomycin 100 U/mL) and maintained at 30.5 °C with 5% carbon dioxide.

### Lentiviral generation

To generate lentiviral particles, HEK293T cells were transfected with pHDM-VSV-G (Addgene#164440), pHDM-Hgpm2 (Addgene #164441), pHDM-Tat1b (Addgene #164442), pRC-CMV-Rev1b (Addgene #164443), and pHAGE packaging vector containing *UBA1* variants in a DNA mass ratio of 1:1:1:1:4, respectively, using Lipofectamine 3000 (Invitrogen). Supernatant was harvested 72 h post-transfection, filtered through a 45 μm syringe-driven filter, and purified overnight using Lenti-X Concentrator (Takara Bio). Concentrated lentiviral pellets were then resuspended in complete CHO ts20 medium and used immediately to infect cells.

### In vivo UBA1 characterization using CHO ts20

Characterization of *UBA1* variants in CHO ts20 cell lines was conducted as previously described [11]. Briefly, *UBA1* variant cell lines were generated by lentiviral transduction with 1.6 μg/mL polybrene using lentiviral particles containing HA-FLAG tagged WT UBA1 and UBA1 variants (M41V, M41T, I50T, Y55H, S56F, L59Q, V75I, G95W, N139K, I301V, C413F, A478S, D585E, N606I, Y618C, D623Y, K746E, R747H, V805I, R869L, I890F, I894S, E1049K). M41V, Y55H, S56F, and A478S were used as positive control, while V75I is a previously tested VUS with intact catalytic activity [21]. After 24 h, lentiviral-containing media was removed and replaced with fresh complete CHO ts20 medium. Transduced ts20 lines were allowed to grow for another 48 h before supplementing media with 5 μg/mL puromycin.

Cells were harvested by trypsinization, counted, pelleted at 300 × *g* for 10 min, media aspirated, and pellet resuspended in complete CHO ts20 medium. Resuspended cells were normalized to one million cells per mL of media. Resuspended cells were then transferred to 1.5 mL microcentrifuge tubes and moved to pre-warmed thermomixers set to 40 °C. Samples were then incubated shaking at 500 rpm for 6 h. Following incubation, heat treated samples were pelleted at 800 × *g* for 10 min, supernatant aspirated, and pellets resuspended in Urea-SDS lysis buffer. Samples were then sonicated for 15 s at 30% amplitude. Sonicated samples were heated at 65 °C for 5 min. Finally, whole cell lysates were separated by SDS polyacrylamide gel electrophoresis and analyzed by immunoblotting. UBA1 blots were run on 6% SDS-PAGE gels to visualize UBA1 isoforms, while all other proteins were resolved using 4–20% gradient SDS-PAGE gels. Immunoblotting blocking was performed with 3% skim milk. Ubiquitylation levels were normalized within each sample to β-actin loading and scaled to WT transfection.

### Proteins, antibodies, and other reagents

Primary antibodies for Poly-ubiquitin (Cell Signaling, 3936S), UBE2D3 (Cell-Signaling, 4330), UBE2L3 (R&D Systems, E2-640), ubiquitylated-H2A (Cell Signaling, 8240), H2A (Cell Signaling, 12349), ubiquitylated-H2B (Cell Signaling, 5546S), H2B (Cell Signaling, 12364), and β-actin (Cell Signaling, 4970) were used at a concentration of 1:1000 and visualized using HRP-conjugated secondary antibodies (anti-rabbit [Cell Signaling, 7074S] or anti-mouse [Cell Signaling, 7076S]) at a concentration of 1:3000. HRP-conjugated antibodies were visualized with Immobilon Western Chemiluminescent HRP Substrate (Millipore, WBKLS0500) or Immobilon ECL Ultra Western HRP Substrate (WBUL0500), depending on signal intensity.

### Reproducibility, quantification, and statistical analysis

Measures taken to verify reproducibility included to perform experiments at least three times. No data was excluded from this study. Densitometry was performed using Fiji. Statistical analyses were performed with GraphPad Prism v.9. Data are presented as mean ± S.D.

## Results

**Fig. 1:**
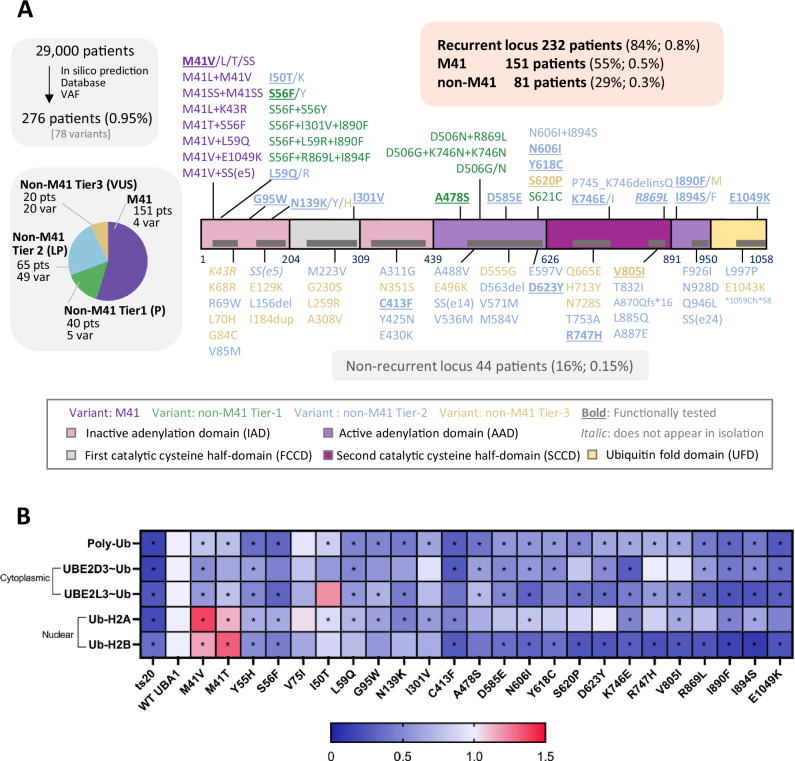
Novel and recurrent *UBA1* variants detected in a screen of 29,000 symptomatic patients. **A** Schematic representation of the *UBA1* gene, highlighting its functional domains and pinpointing the exact locations of the identified *UBA1* variants. The variants were classified into Tier-1 (pathogenic: P), Tier-2 (likely pathogenic: LP) and Tier-3 (variant of uncertain significance: VUS), as depicted in the outer pie chart on the left. *UBA1* variants are displayed on top of the gene if the locus occurred recurrently in our screen. Combination of variants detected in the same patients are also shown. Variants detected only as combinations are italicized. M41 and other tier assignments are color-coded. Bold underlined variants were tested for functional significance. **B** Chart summarizing *UBA1* variants and their functional status, indicating the presence or absence of ubiquitylation. Quantification of polyubiquitin levels and mono-ubiquitylated histone H2A/B (Ub-H2A, Ub-H2B) or E2 enzymes (UBE2D3-Ub, UBE2L3-Ub) were normalized within each sample to β-actin levels and scaled to WT transfection. E2 enzyme ubiquitylation was quantified as the ratio of charged form to uncharged form and scaled to WT. Data represent n = 3–6 biological replicates, shown as mean −/+ s.d., significance determined by unpaired t-test with Welch’s correction (*p < 0.05, **p < 0.01).

Between July 2022 and September 2024, *UBA1* sequencing, as part of the myeloid panel, was performed on 31,554 samples of 29,000 unique patients. A total of 276 predominantly male patients (97% male) were identified with *UBA1* M41, non-M41 P/LP variants or non-M41 VUS (Fig. 1A). Fourteen patients carried multiple variants. M41 variants (M41T [N = 69], M41L [N = 34], M41V [N = 33], M41ss [N = 16]) occurred at the highest frequencies, and 18 non-M41 loci (14/18 novel) also occurred recurrently, with the known S56F (N = 15) and A478S (N = 14) most frequent (Supplementary Table 3).

As most known pathogenic variants were recurrent, we tested all 14 recurrent loci for functional validation. In addition, we examined 4 from the 46 non-recurrent variants. We used WT *UBA1* and a known benign VUS V75I as negative controls for experiments, with M41V, M41T, Y55H, S56F, A478S serving as a positive control based on our previous studies. Functional read-outs included global polyubiquitylation and H2A/B monoubiquitylation, which have been used as an indicator of UBA1 functional impairment [22–25], and E2 enzyme charging (UBE2L3/UBE2D3) [3]. All variants caused dysfunction with respect to global ubiquitylation levels and various degree of other ubiquitylation defects (Fig. 1B, Supplementary Fig. S1). Interestingly, M41 variants showed an increase in H2A/B monoubiquitylation, whereas non-M41 variants showed decreased nuclear ubiquitylation. As all variants were functionally defective, in the following analyses, we treated the 81 patients harboring variants in recurrent loci as non-M41 variants to contrast with the M41 patients.

### UBA1 M41 and non-41 mutations show distinct co-mutation patterns

**Fig. 2:**
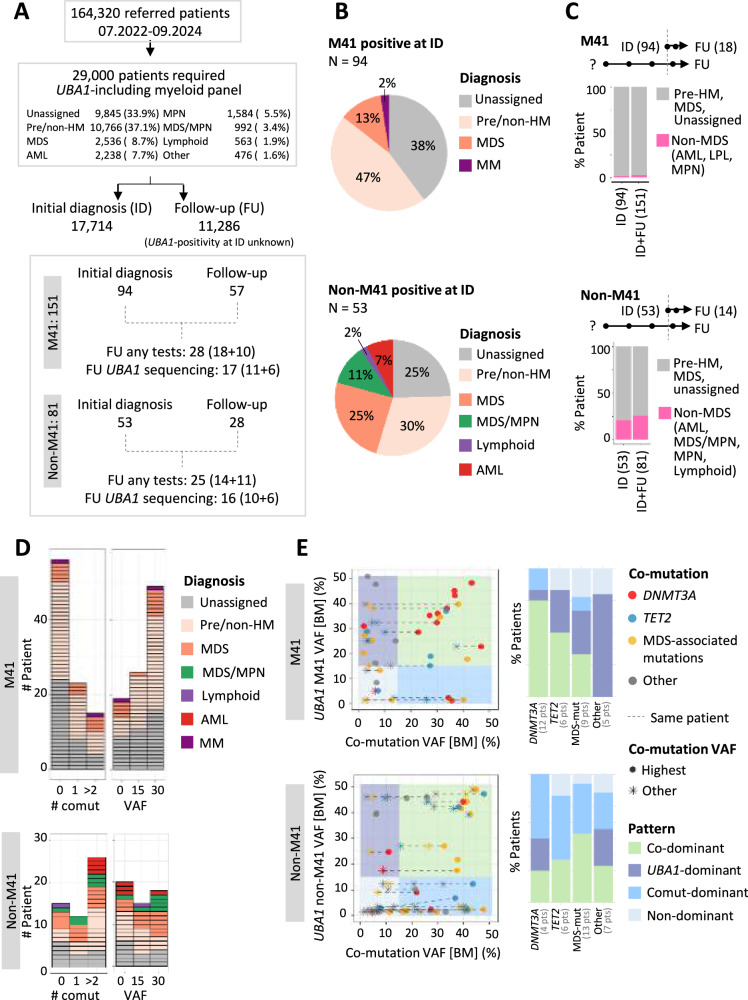
Distinct oncologic and clonal characteristics of *UBA1* M41 and non-M41 mutations. **A** Patients who underwent *UBA1*-including 62 myeloid gene panel were included in the study. Patients were classified into those referred as initial diagnosis and those as follow-up. A subset of patients underwent further follow-up for diagnostic tests, including *UBA1*-sequencing. The parenthesis shows the composition of follow-up samples (number of ID patients + number of patients with FU sample only). **B** Diagnosis of patients with *UBA1* M41 and non-M41 variants at initial diagnosis. **C** The proportion of non-MDS malignant diagnosis of all patients (ID + FU) at the end of the enrollment period. Number of ID patients and FU samples of ID patients by the end of enrollment period are shown on top. For patients with multiple follow-up samples, the diagnosis of the samples at the latest time point is shown. **D** The distribution of VAF and co-mutation status of patients at initial diagnosis. **E** The relationship of VAF between *UBA1* and co-mutations. The left-side panels show the VAF of *UBA1* and co-mutations, separated by M41 and non-M41. Samples with multiple mutations are indicated by dots connected with dashed lines. The right-side bar plots categorize patients by type of co-mutation and illustrate the patterns of clonal dominance, defined as follows: co-dominant (both VAFs > 15%), *UBA1*-dominant (*UBA1* VAF ≥ 15% and co-mutation VAF < 15%), co-mutation dominant (*UBA1* VAF < 15% and co-mutation VAF ≥ 15%), and non-dominant (both VAFs < 15%). Comut: co-mutation. VAF: variant allele fraction. VAF is halved for all males. MDS-mut: *ASXL1*, *BCOR*, *EZH2*, *STAG2*, *SF3B1*, *SRSF2*, *ZRSR2*, *U2AF1*.

Our cohort consisted of symptomatic patients who underwent myeloid gene panel sequencing as part of the diagnostic work-up and included patients diagnosed with various hematologic malignancies, particularly myeloid malignancies. We divided the cohort into patients whose *UBA1* mutation status was known at initial diagnosis (ID) and those who had *UBA1* sequencing during follow-up in various disease stages or after treatment (FU) (Fig. 2A). We detected M41 mutations in 94 patients (M41 patients) and non-M41 mutations in 53 patients (non-M41 patients) at ID.

As we selected patients based on the presence of genetic information, a definite hematologic diagnosis was not available for all patients. An MDS diagnosis was given in 13% of M41 positive cases and in 25% of non-M41 cases (Fig. 2B). The predominant MDS subtype for M41 patients was MDS-LB (50%), followed by MDS-IB1 (25%), MDS-RS (17%), and MDS-*SF3B1* (8%), while for non-M41 patients it was MDS-IB1 (38%), then MDS-RS (31%), MDS-LB (23%), and unspecified (8%). We observed a ten times higher proportion of non-MDS malignant diagnosis (20%; AML 7%, Lymphoid [Mature B cell neoplasm] 2%, MDS/MPN 11%) for the non-M41 patients compared to M41 patients (MM 2%) at ID (Fig. 2B, chi-square test p < 0.05).

We were also interested in the difference in risk of progression. Our data collection depended on the necessity of follow-up information of the physicians who send us samples, which introduces the bias that stable patients will not be followed up. Acknowledging this limitation and assuming that stable patients were not followed up, we compared the diagnosis at ID and diagnosis at the end of the enrollment period including patients whose *UBA1* status was unknown at ID and allowing heterogeneity in length of follow-up. M41 patients remained largely non-MDS, whereas non-M41 patients had slightly increased proportion of patients diagnosed with non-MDS malignant entities (Fig. 2C). In terms of risk of progression, we observed that one MDS patient (M41V) progressed to AML in one year, and one MPN and one lymphoplasmacytic lymphoma patient had M41V detected at FU. For non-M41 patients, limiting to progression cases (changes in diagnoses), four progressed to AML from pre-HM or CMML over the courses of 2.5-, 4-, 6-, and 8 years, and two progressed to CMML from pre-HM within a year (Supplementary Fig. S2).

The comparative analysis of inflammation between *UBA1* M41 and non-M41 patients is complicated by the hematology-based referral of our cohort. As a result, clinical records often lack uniformity and detail in documenting inflammation. However, a review of the available clinical records for non-M41 patients revealed potential inflammatory manifestations in 9 patients (11%), including skin symptoms (vasculitis [P154:S56F + I301V + I890F, P232:I894S + N606I], neutrophilic dermatosis or Sweet Syndrome [P173:A478S, P198:L59Q], unspecified skin inflammation [P223:N606I]), suspected paraneoplastic pneumonitis [P170:A478S], unspecified rheumatic disease treated with long-term steroids [P219:K746E], the coexistence of rheumatoid arthritis and polymyalgia rheumatica [P213:S56Y] and unspecified VEXAS-like symptoms [P177:D506N]. These findings indicate that patients with non-M41 mutations can show clinical characteristics of VEXAS syndrome.

Next, we analyzed the distribution of VAF and number of co-mutations. M41 mutations were most frequently the dominant and isolated mutations, whereas non-M41 mutations occurred more often with co-mutations and at various VAFs (Fig. 2D). Analysis of BM (N = 116) versus PB (N = 31) samples (Supplementary Fig. S3A) as well as ID + FU samples (Supplementary Fig. S3B) yielded consistent findings, except in PB the VAF tended to be lower. In fact, for 7 patients for whom we had both BM and PB samples within 2 months, BM showed higher VAF than PB with two samples (M41 N = 1, non-M41 N = 1) negative in PB (Supplementary Fig. S4).

In our analysis of co-mutation partners and their clonal relationships, *DNMT3A* mutations emerged as the most frequent co-mutations for M41 mutations (38%, N = 12), 75% of which were co-dominant with *UBA1* mutations (Fig. 2E). Conversely, non-M41 mutations were predominantly associated with non-*DNMT3A* mutations, particularly MDS-associated mutations (Fig. 2E) as co-dominant co-mutations. This pattern was confirmed upon extending the analysis to all patients with co-mutations (M41 N = 59; non-M41 N = 58) (Supplementary Fig. S5). Additionally, among M41 patients, the VAF of isolated M41 variants was higher than those with co-mutations (31.9% vs. 24.5%, p < 0.05, Wilcoxon test).

In 33 patients (M41 N = 17, non-M41 N = 16) analyzed for *UBA1* over multiple time points, we examined the interaction between M41 and non-M41 mutations with other genetic aberrations over time and in response to treatment (Supplementary Fig. S6). Although this analysis is limited by the small, potentially biased subset of patients with multiple sequencings, overall, no differences were observed in treatment response to azacitidine, other chemotherapies, or HSCT. Both groups showed relatively stable VAF when no hematologically significant treatment was given.

### UBA1 mutations in comparison to established CHIP and MDS associated variants

**Fig. 3:**
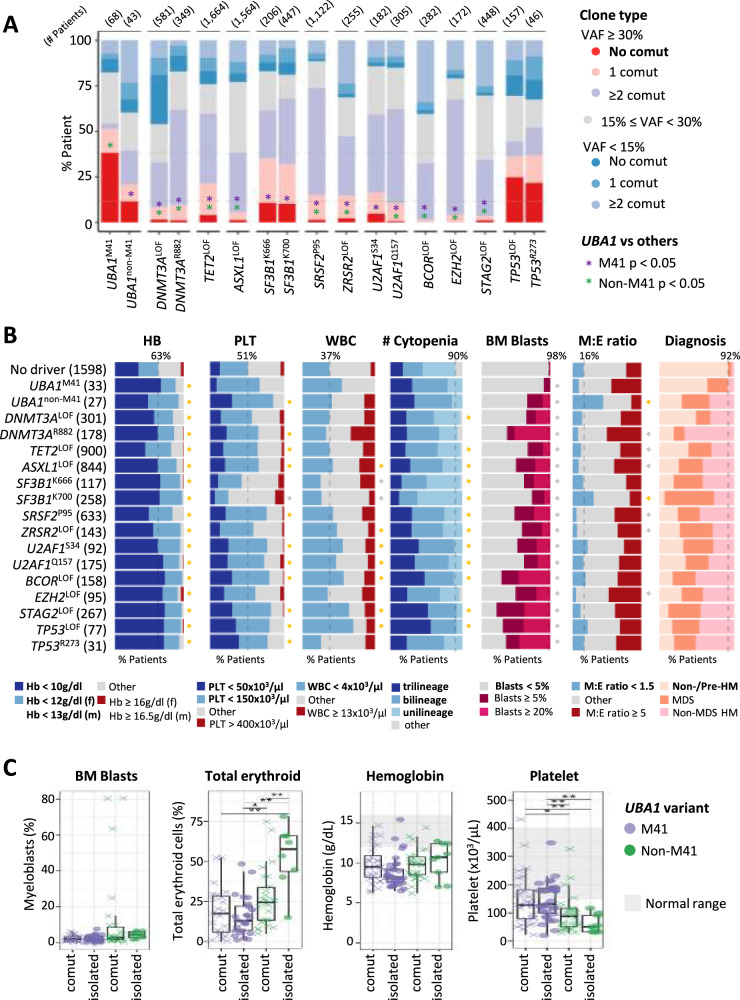
*UBA1* variants in comparison to specific CHIP- and MDS-associated variants. **A** Proportion of patients with independent large clones among patients at initial diagnosis stage evaluated with BM samples. Numbers in parenthesis on top indicate the number of patients. Fisher’s tests corrected by the Benjamin-Hochberg method are performed for both M41 and non-M41 variants against all other variants for statistically different proportion of patients with large independent clones (no co-mutation VAF > 30%). * p < 0.05. **B** Hematologic phenotypes of *UBA1* variants compared to specific CHIP- and MDS-associated variants of patients at ID with BM samples and with morphologic parameters available, compared to the group of patients presenting with cytopenia or symptoms not otherwise specified without genetic driver (Cytopenia/NOS - no driver), labeled no driver. Proportion of patients satisfying conditions specified in the color code (bold) are shown. Dashed line shows the proportion of a selected condition of the reference. Fisher’s tests corrected by the Benjamin-Hochberg method are performed. f: female, m: male. * p < 0.05. Color of the * indicates statistical higher (yellow) or lower (gray) proportion. **C** Comparison of BM and blood counts of *UBA1* M41 and non-M41 variants in isolation and with co-mutations. Wilcoxon rank sum test corrected by the Benjamini-Hochberg method was performed. * p < 0.05, ** p < 0.01.

To place the clonal behavior of *UBA1* mutations into context, we compared the clonal architecture of *UBA1* mutations with specific variants associated with CHIP and MDS. As treatment and material differences affect VAF, we compared data from the BM samples at ID (N = 11,052). The results of the analysis showed that M41 mutations uniquely exhibit a high proportion of large clones (VAF ≥ 30%) without co-mutations, even in comparison to variants commonly associated with CHIP and MDS (Fig. 3A). While not as prominent as with M41 mutations, non-M41 mutations also showed a significant number of patients, who carried *UBA1* as a sole mutation with high VAF.

We next tried to understand the hematologic phenotype of *UBA1* variants in comparison to selected CHIP/MDS variants (Fig. 3B, Supplementary Fig. S7). To characterize the hematologic phenotype of each mutation, we compared their BM and blood counts with a group constructed by taking symptomatic patients with no genetic aberrations detected. These patients had cytopenia or hematologic symptoms not otherwise specified (Cytopenia/NOS—no driver). Non-M41 mutations were characterized by decreased M:E ratio, similar to the *SF3B1* K700 variant but with a decrease in platelet counts (Fisher’s test, p < 0.05, Fig. 3B). A review of clinical records for 81 non-M41 patients identified 5 patients with documented signs of hemolysis (Coombs negative hemolysis or laboratory results with low haptoglobin, high lactose dehydrogenase and hyperbilirubinemia) and 9 additional patients with hyperbilirubinemia only, which may suggest increased erythropoiesis in response to peripheral hemolysis. Among the 5 patients with documented hemolysis, 3 had no other co-mutations and no inflammatory symptoms (P155:S621C, P179:A478S, P201:I301V).

As *UBA1* mutations can occur both either as a sole mutation or together with co-mutations, we investigated if the unique phenotype arises from isolated mutations or is a result of co-mutations (Fig. 3C). There was no difference in the hematologic phenotype between isolated or co-mutated M41, probably since the co-mutations are non-dominant. On the other hand, increased erythropoiesis was most pronounced in isolated non-M41 mutations. All groups commonly presented with anemia and thrombocytopenia, yet non-M41 patients, particularly isolated, exhibited deeper thrombocytopenia. Thus, *UBA1* non-M41 mutations seem to have a distinct phenotype in isolation than with co-mutations.

## Discussion

In this study, we present the largest and most rigorously controlled cohort to date, with both genetic and morphologic examinations evaluated at a single institution. We found that *UBA1* M41 and non-M41 variants exhibit distinct hemato-oncologic and biochemical profiles, indicating distinct pathology.

In our comprehensive study, we sequenced 29,000 patients diagnosed with a variety of hematologic malignancies and identified approximately 80 *UBA1* variants that are rare or virtually absent in the healthy population. To assess their impact, we validated variants in all of the previously unvalidated recurrent loci and 4 non-recurrent variants, all of which exhibited significant defects in the ubiquitylation process. Furthermore, almost all of the loci (16/18) on which we recurrently detected variants have been reported in other hematology cohorts [5, 7–10] as well as 15% of the non-recurrent loci (L70H, G84C, C413F, E496K, E597A/V, R747H/C, N928D). The enrichment of *UBA1* functional variants in patients demonstrating hematologic manifestations underscores the critical role of functional *UBA1* in normal differentiation and blood production.

Our analysis revealed that the two classes of *UBA1* variants—M41 and non-M41—result in distinct alterations in nuclear ubiquitylation tested by measuring H2A/B monoubiquitylation pattern. M41 variants have preserved nuclear UBA1a activity, whereas non-M41 variants have impaired UBA1a activity leading to this discrepancy in activity. This difference may underline distinct pathophysiological mechanisms. In fact, in contrast to the known myeloid bias of M41 variants [2], the S56F variant has been reported to show increased erythropoiesis in multiple case reports [26, 27] and a case series (N = 6) [6]. In addition, patients harboring non-M41 variants (N = 5) in the active adenylation domain were reported to manifest with erythroid hyperplasia and signs of hemolysis [5]. Our previous screening also included 3 hemolytic patients out of 16 non-M41 patients [7]. This larger study confirmed the high proportion of erythroid cells at initial diagnosis of non-M41 patients compared to patients without genetic aberrations, particularly in the absence of co-mutations, and reported 5 additional cases with hemolytic anemia. This difference prompts further investigation into whether different approaches to managing cytopenias might be warranted for patients with M41 versus non-M41 mutations. For example, if diagnosed with MDS, patients carrying isolated *UBA1* mutations classify as low-risk MDS, for which erythroid stimulating agents (ESA) and luspatercept, an erythroid maturation agent, may be used to manage anemia [28]. Both treatments are considered to be more effective in the presence of specific erythroid precursors [29, 30]. In this regard the non-M41 mutations may have a higher chance of response than M41 mutations due to the substantial presence of potentially responsive erythroid progenitors. Luspatercept has also been approved for β-thalassemia [31], which is a type of hemolytic anemia, and may have analogous effect in hemolytic non-M41 patients. Indeed, a recent study found higher response rate of luspatercept among patients carrying the non-canonical *UBA1* variants [32]. Understanding these nuances is essential for developing pathobiology-based effective treatments for affected individuals.

Our study also highlights the distinct clonal behavior of M41 mutations in comparison to both non-M41 mutations and other well-known CHIP/MDS variants. We confirm the paucity of co-mutations, which is often limited to *DNMT3A* mutations, echoing findings from a previous study (N = 80) which investigated co-mutations in 39 genes in peripheral blood of patients already diagnosed with VEXAS [33]. With 151 M41 patients analyzed using a 62-gene myeloid panel, our cohort represents the largest group studied to date, encompassing a range of hematologic malignancies, including AML. The observed pattern underscores the low-risk nature of M41 mutations and aligns with preclinical evidence suggesting the anti-leukemia efficacy of *UBA1* inhibitors [34, 35].

Furthermore, our analysis revealed a high proportion of patients with isolated large *UBA1* M41 clones, and we show that this is a characteristic not observed in patients with typical CHIP/MDS variants. Recent studies have shown that the clonal fitness of M41 variants comes from their ability to withstand the toxic inflammatory microenvironment [36]. In addition, multiple evidence suggests that the mere presence of the *UBA1* mutations does not confer inherent proliferative capacity [37, 38], which is again quite characteristic of MDS-associated mutations [39–41]. However, in comparison to CHIP/MDS-associated mutations *UBA1* M41 mutations do not gain clonal advantage by accumulating additional mutations, which probably confers its characteristic low risk. This finding suggests that VEXAS constitutes a distinct molecularly defined entity different from CHIP/CCUS/MDS.

Conversely, the role of non-M41 mutations and their co-mutation patterns have been less clear. Our study shows that while the frequency of large clones without co-mutations in non-M41 patients is lower than in M41 patients, it is higher than in patients with other established variants, except for those with *SF3B1* and *TP53* mutations. Concerning the role of inflammation in this aspect, a recent British study showed that at an individual with an A478S mutation and four additional co-mutations developed VEXAS symptoms [10]. All our inflammatory A478S patients also had co-mutations (Supplementary Table 4), whereas the patient carrying isolated A478S was not inflammatory but severely hemolytic, suggesting VEXAS-like inflammation may indicate acquisition of co-mutations in some contexts.

In conclusion, our study underscores the pathogenicity of *UBA1* variants, revealing that both M41 and non-M41 variants hold prognostic and therapeutic potential. We confirmed the functional relevance of non-M41 variants and delineated the distinct hematologic phenotype and co-mutation spectrum associated with M41 variants. This establishes the unique role of M41 variants, especially in relation to VEXAS and VEXAS MDS entities. However, the impact of non-M41 variants on clone size and co-mutation profiles across various entities requires further exploration to fully understand their clinical implications.

## Supplementary information

Supplemental material

## Data Availability

All data analysed during this study are included in this published article and its supplementary information files.
