# Supplementary material for: Distinct characteristics of VEXAS-causative UBA1 M41 and recurrent functional non-M41 mutations
Source: Leukemia. 2025 Oct 9;39(12):2872–80. doi: 10.1038/s41375-025-02775-4 (PMC12634433; doi:10.1038/s41375-025-02775-4)
Supplement: Supplementary file 1 — Supplemental material [file 41375_2025_2775_MOESM1_ESM.pdf]

Supplemental material for

Distinct characteristics of VEXAS-causative *UBA1* M41 and recurrent functional non-M41 mutations

Maki Sakuma[1,2*], Amy K. Wang[3*], Samuel J. Magaziner[3], Sachiko P. Keane[3], Manja Meggendorfer[1], Wolfgang Kern[1], Claudia Haferlach[1], Torsten Haferlach[1], David B. Beck[3,4,5*], Wencke Walter[1*†]

[1] Munich Leukemia Laboratory, Munich, Germany

[2] Graduate School of Medicine and Health, Technical University of Munich, Munich, Germany

[3] Center for Human Genetics and Genomics, NYU School of Medicine, New York, NY, USA

[4] Department of Medicine, NYU School of Medicine, New York, NY, USA

[5] Department of Biochemistry and Molecular Pharmacology, NYU School of Medicine, New York, NY, USA

* These authors contributed equally

†Corresponding Author. E-Mail: wencke.walter@mll.com

This PDF file includes:

Figures S1 to S7

Tables S1 to S4

Supplementary Figures and Legends

Figure S1A.

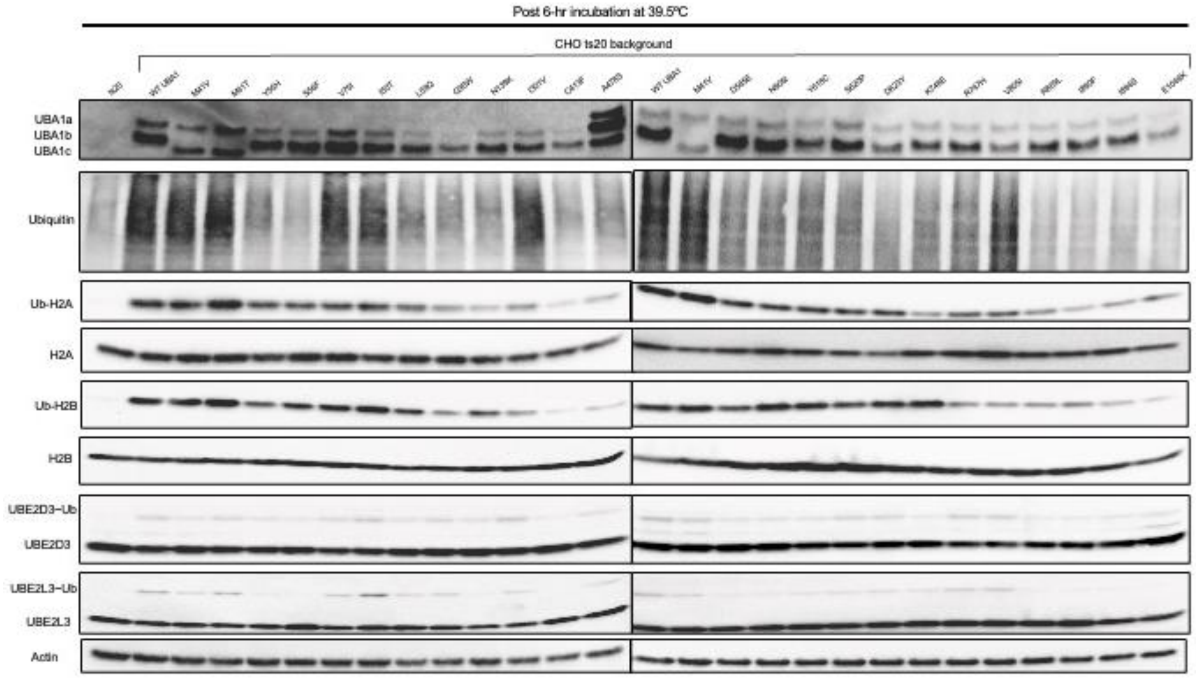

Supplementary Figure S1A. Representative Western blot analysis of the ubiquitylation defects seen in *UBA1* variants. Chinese hamster ovary (CHO) cells carrying a temperature-sensitive *UBA1* allele were lentivirally transfected with the indicated *UBA1* variants. Cells were incubated at the restrictive temperature (39.5ºC) for 6 hours, followed by immunoblotting for UBA1, polyubiquitin, ubiquityl-histone H2A/B, ubiquityl-UBE2D3, ubiquityl-UBE2L3 and actin. Ponceau S staining is included as a loading control to confirm equal protein loading across all samples. Polyubiquitylation was rescued with WT UBA1 and the benign mutation (V75I).

Figure S1B.

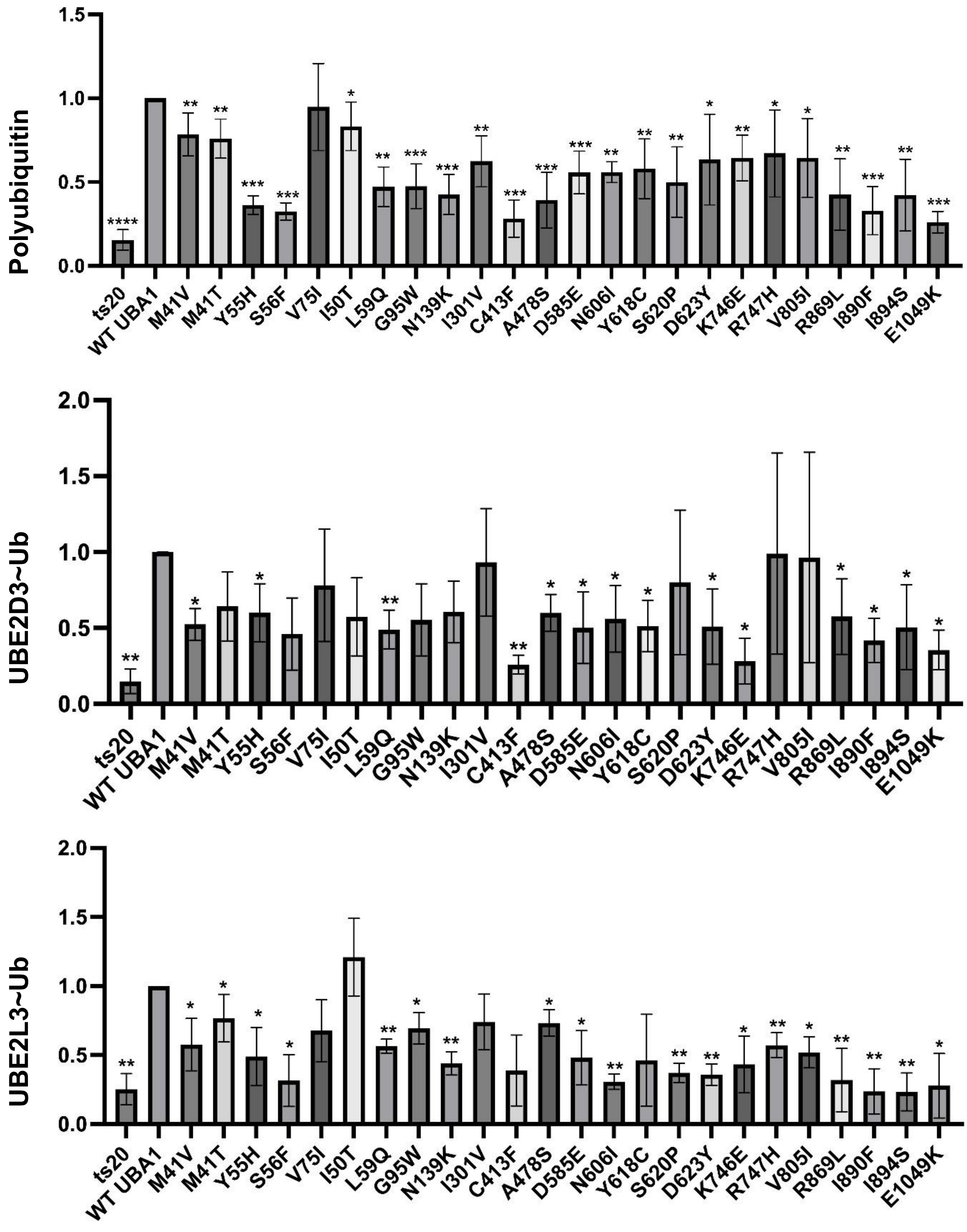
Polyubiquitin
1.5
1.0
0.5
0.0
ts20
WT UBA1
M41V
M41T
Y55H
S56F
V75I
I50T
L59Q
G95W
N139K
I301V
C413F
A478S
D585E
N606I
Y618C
S620P
D623Y
K746E
R747H
V805I
R869L
I890F
I894S
E1049K
UBE2D3~Ub
2.0
1.5
1.0
0.5
0.0
UBE2L3~Ub
2.0
1.5
1.0
0.5
0.0

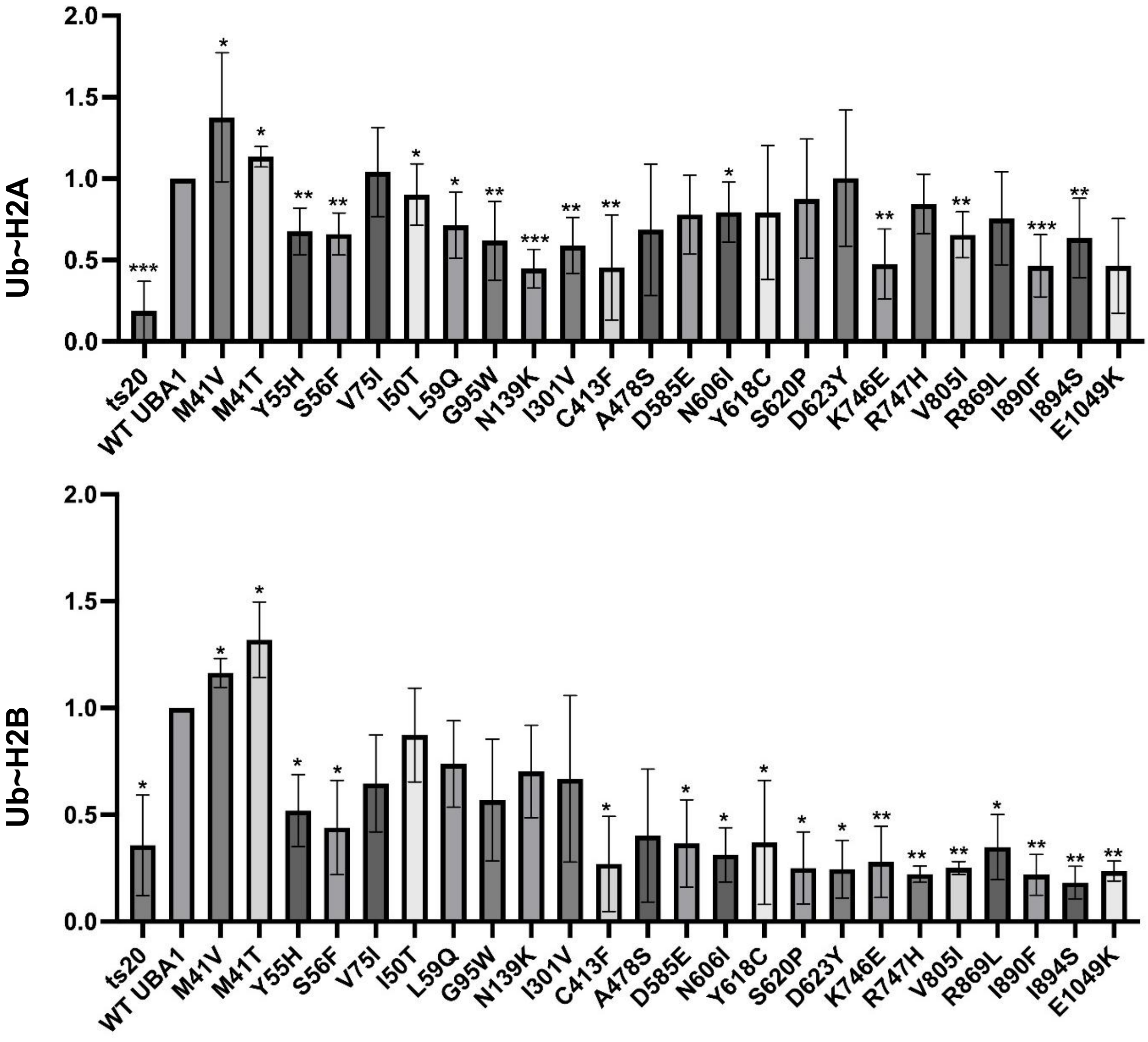

Supplementary Figure S1B. Quantification of the ubiquitylation defects in *UBA1* variants. Ubiquitylated substrates were normalized to β-actin expression level and then to WT UBA1. Data represent n = 3-6 biological replicates, shown as mean −/+ s.d., significance determined by unpaired t-test with Welch's correction (*p < 0.05, **p < 0.01).

Figure S2.

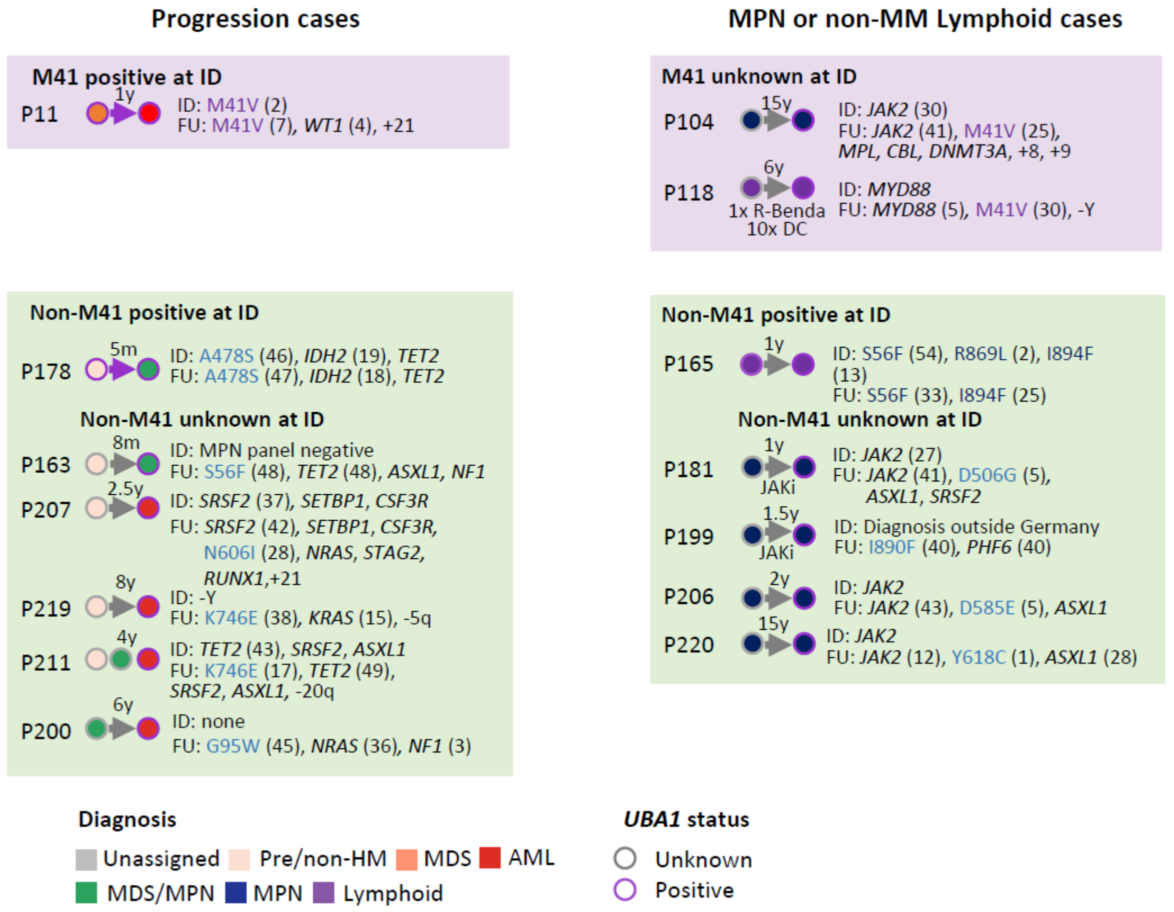

Supplementary Figure S2. Progression and other atypical cases. For each patient, the diagnosis (color-coded) and variants detected at initial diagnosis (ID) and at follow-up (FU) are shown. The VAF of the variants are shown inside the parenthesis. If known, chromosomal aberrations are shown after the variants. The protein-level change variants are shown for *UBA1* instead of the gene name and is colored. If known, the treatment received is given under the arrow. The duration between datapoints is shown above the arrow. When more than one year, duration is shown in increments of 0.5 years. y: years, m: months. 1x R-Benda: one cycle of Rituximab-Bendamustin, 10x DC: ten cycles of Dexamethasone and Cyclophosphamide. JAKi: JAK inhibitors.

Figure S3.

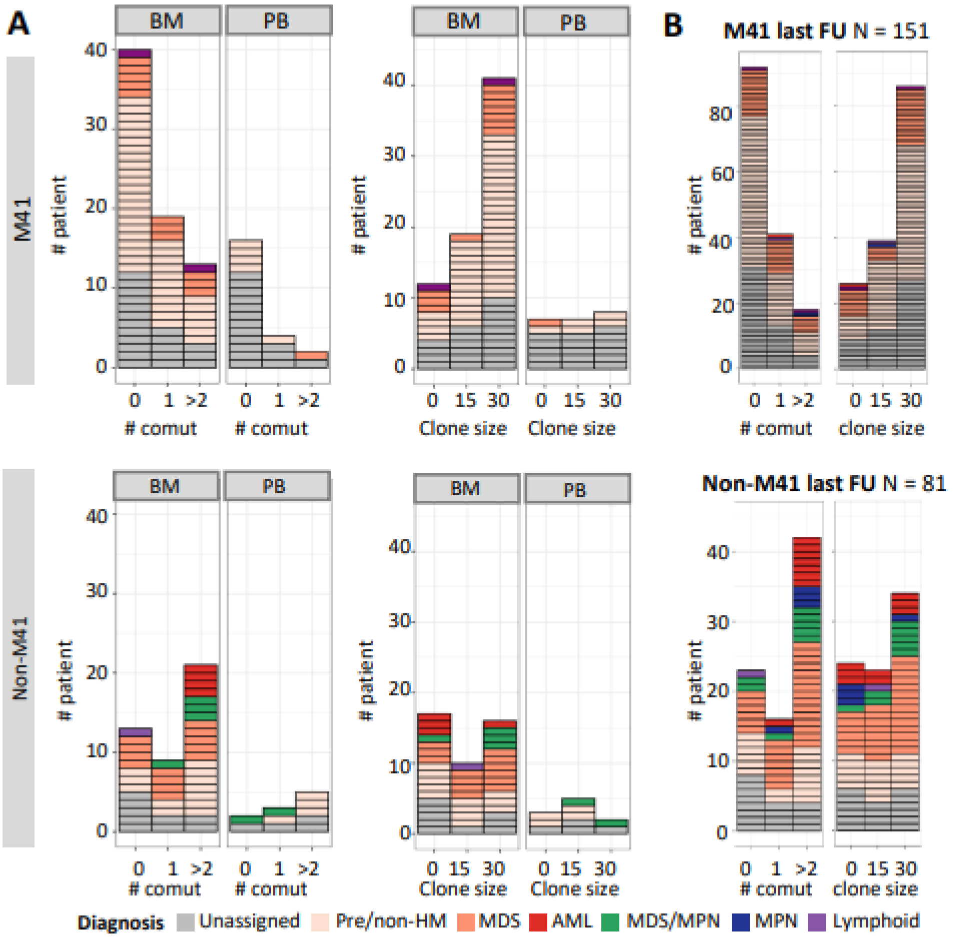

Supplementary Figure S3. The distribution of patients by co-mutations and VAF levels of various subsets. **(A)** The distributions of initial diagnosis (ID) samples separated by bone marrow (BM) or peripheral blood samples (PB). **(B)** The distribution of initial diagnosis and follow-up samples combined. For patients with multiple datapoints the sample at last follow-up with the most complete information is taken.

Figure S4.

A

Variant	Patient ID	PB	BM
M41	P47	negative	positive
M41	P103	positive	positive
M41	P87	positive	positive
M41	P102	positive	positive
Non-M41	P228	negative	positive
Non-M41	P172	positive	positive
Non-M41	P223	positive	positive

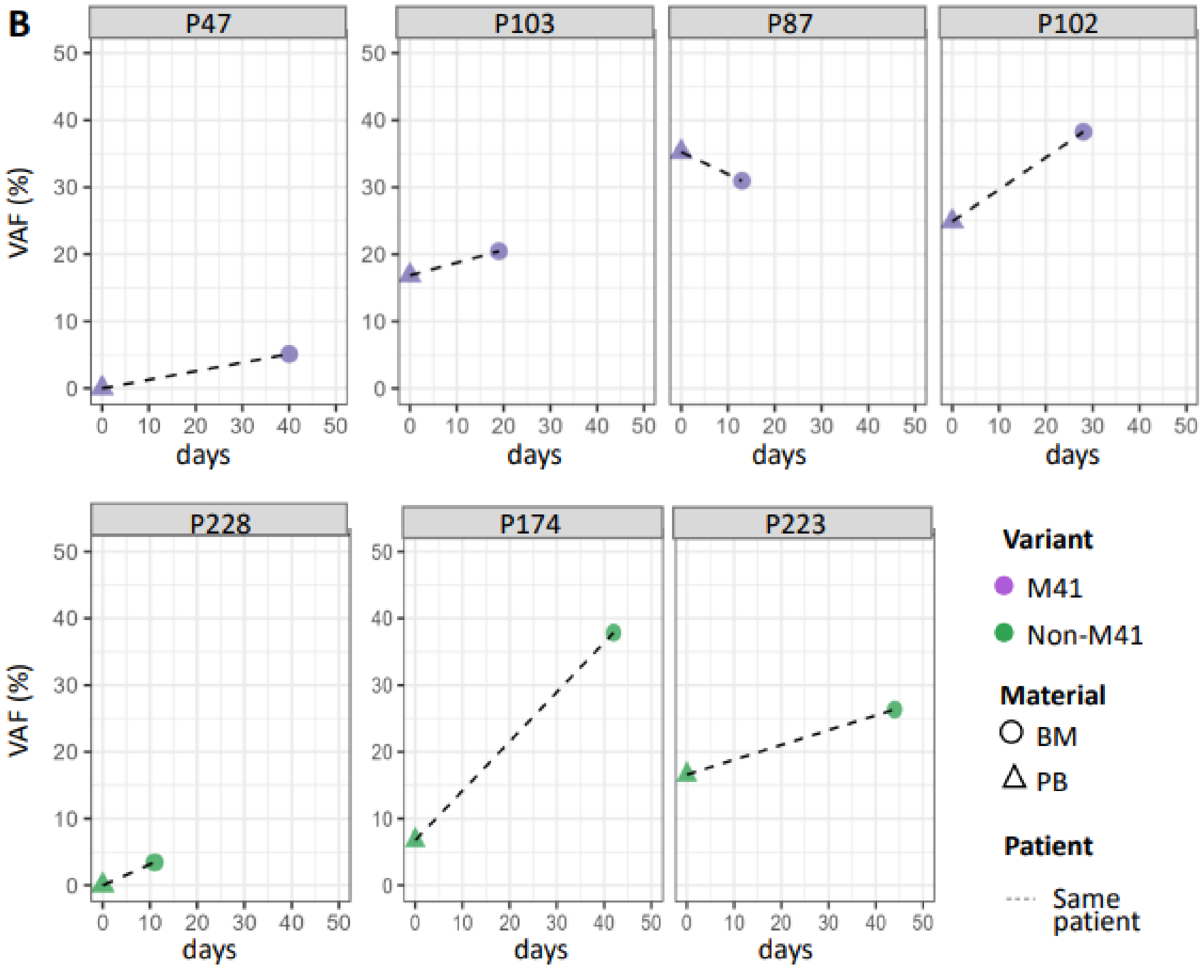

Supplementary Figure S4. *UBA1* VAF difference between BM and PB samples from the same patient during initial diagnosis. (**A**) *UBA1* variant positivity by patient. (**B**) VAF of each patient with both BM and PB samples is shown by timepoints. The left most panel shows cases for which peripheral blood was UBA1 negative. The right panels show cases for which both PB and BM were positive for *UBA1*. 0: VAF < 15%, 15: 15% ≤ VAF < 30%, 30: 30% ≤ VAF ≤ 50%. VAF: variant allele fraction. BM: Bone marrow. PB: peripheral blood

Figure S5.

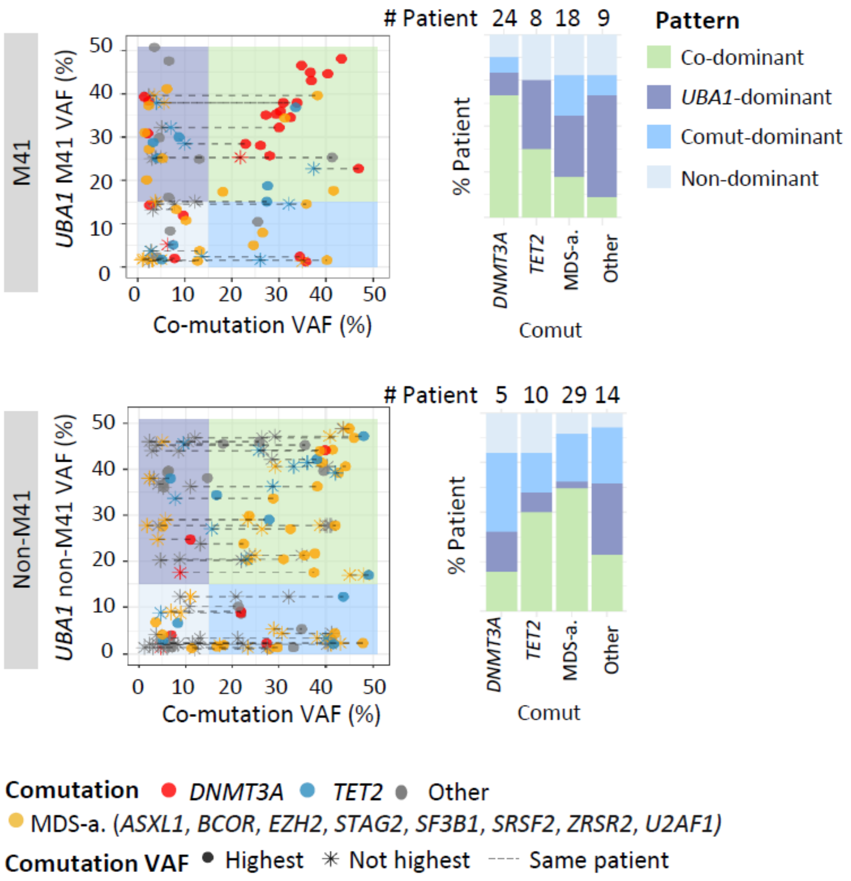

Supplementary Figure S5. Distinct clonal architecture of M41 and non-M41 variants with all patients. The left-side panels show the VAF of *UBA1* and co-mutation. Samples demonstrating multiple mutations are represented with multiple dots connected with dashed lines. The co-mutation with highest VAF is represented in the right-side panels. In the right-side panels, patients were separated in groups by co-mutation type (absolute number shown on top of the figure) and the proportion of their clonal dominance pattern are shown. co-dominant (both VAF > 15%), UBA1-dominant (UBA1 VAF ≥ 15%, co-mutation VAF < 15%), co-mutation dominant (UBA1 VAF < 15%, co-mutation VAF ≥ 15%) and non-dominant (both VAF < 15%). Comut: co-mutation. VAF: variant allele fraction. VAF is halved for all males.

Figure S6.

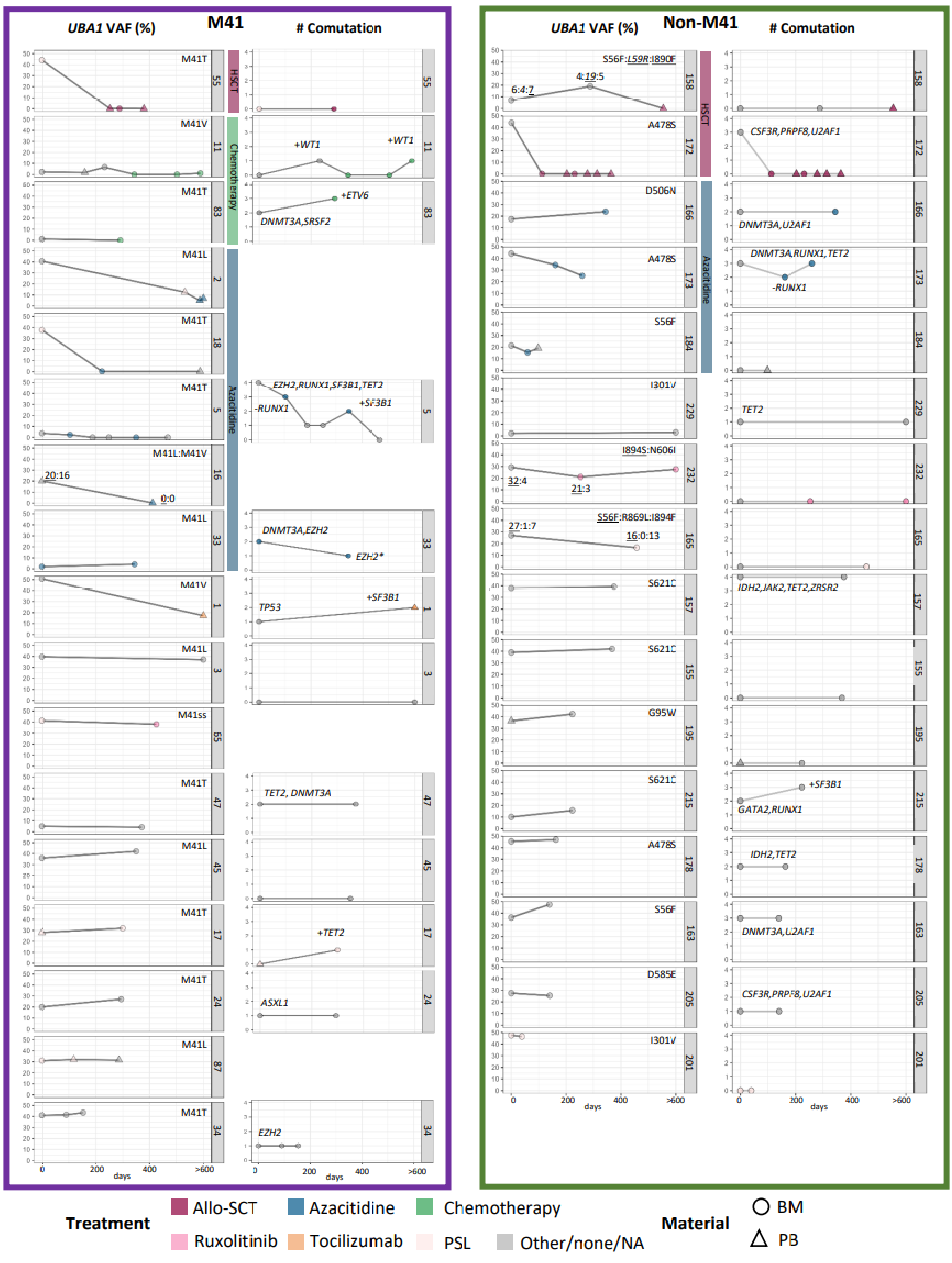

Supplementary Figure S6. Longitudinal tracking of VAF (left) and number of co-mutations in patients with multiple sequencing results separated by M41 (purple box)

and non-M41 (green box) patients. Dots (round: BM; triangular: PB) represent the time points the samples were sent for sequencing. M41 patients without co-mutation tracking figure indicates that only *UBA1* sequencing was performed for the later timepoints. For patients with multiple *UBA1* mutations the VAF of the variants are supplemented by text in the same order as shown at the top right, and only the one with the highest VAF is plotted and the VAF of the other mutations are given as text annotations. For the co-mutations, the genes which were positive are specified as text on the side of the dots. A plus sign (+) suggests that the co-mutation was gained compared to the last timepoint. A minus sign (-) suggests that the co-mutation was lost compared to the last timepoint. P33 *EZH2** denotes that the previously positive *DNMT3A* was not sequenced and does not indicate loss of *DNMT3A*. Treatment initiated between the two timepoints are color-coded in the later timepoint. In most cases the exact treatment start timepoint is not communicated to MLL from the referring physicians. Time points after 600 days from the first sample are coded as 600 days. Gain and loss of co-mutations are color-coded in red and blue respectively. Allo-SCT: allogenic stem cell transplantion. PSL: Prednisolone.

Figure S7.

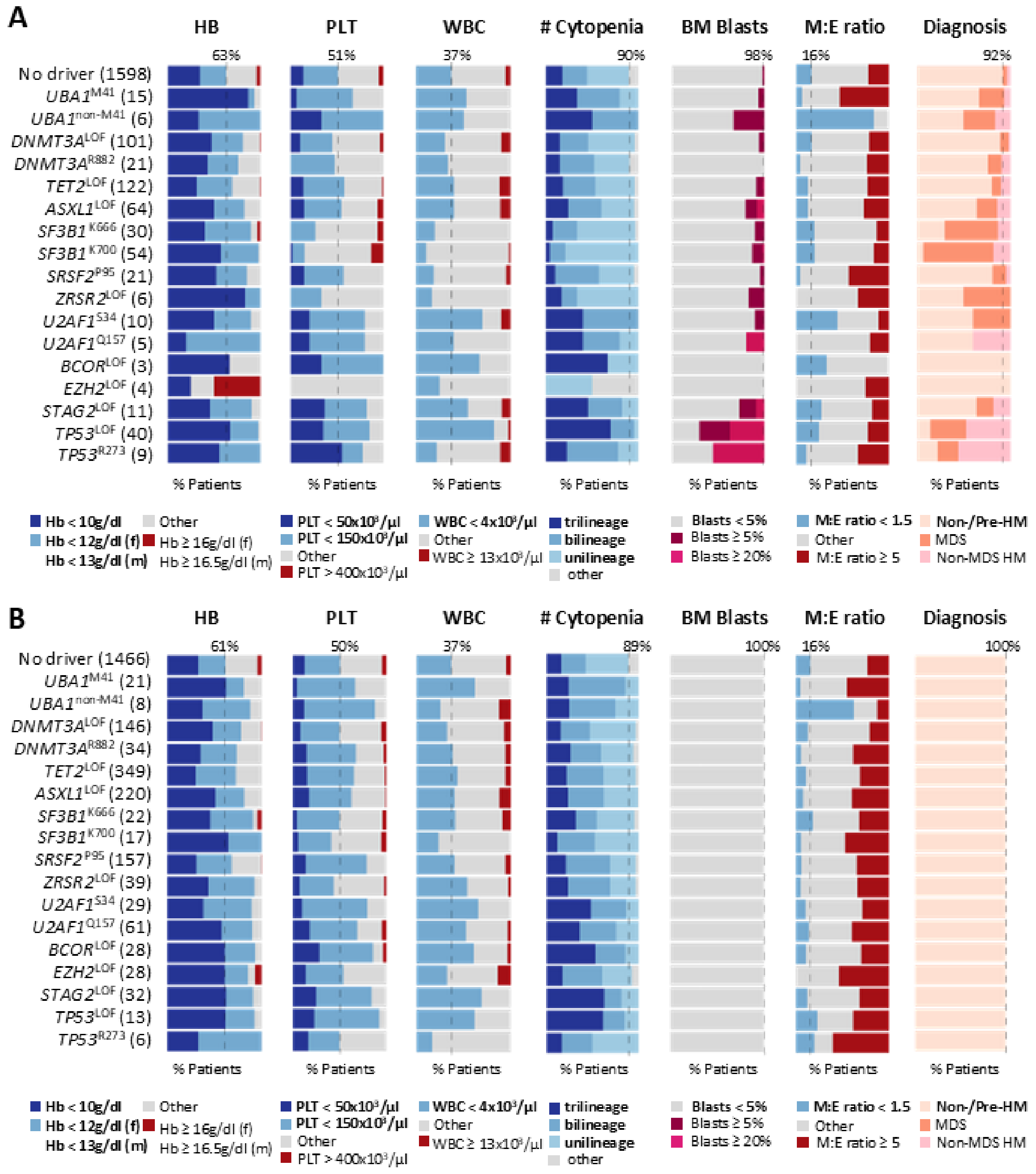

Supplementary Figure S7. Hematologic phenotypes of *UBA1* variants and specific CHIP-/MDS-associated variants of patients at ID with BM samples compared to the reference (No driver: Cytopenia/NOS). Patients limited to those harboring isolated mutations (**A**) and limited to those without malignant diagnoses (**B**) are shown respectively. Proportions of patients satisfying conditions specified in the color code are shown. Dashed line shows the proportion of the condition in bold on the legend of the reference. The numbers next to each variant indicate the number of patients harboring the corresponding variant. Statistical tests were not performed. M:E ratio: myeloid-to-erythroid ratio. BM bone marrow. f: female, m: male.

Supplementary Tables

Supplementary Table 1

		ID	ID-FU	FU	FU-FU	No-UBA1
Total		17714	4387	11286	3138	135320
Material						
BM		11052	NR	NR	NR	NR
PB		6261	NR	NR	NR	NR
Other		401	NR	NR	NR	NR
Sex						
Male		9903	2582	6489	1894	NR
Female		7811	1805	4797	1244	NR
Diagnosis						
All	Treated	0	732	315	568	6983
All	AML	1857	317	381	166	1057
All	MDS	1725	387	811	236	1140
All	MDS/MPN	602	216	390	157	486
All	MPN	436	64	1148	152	3917
All	Lymphoid	186	69	197	50	13733
All	MM	79	35	101	14	4280
All	Non/Pre-HM	7458	937	3308	552	49003
All	Other HM	118	19	43	15	408
All	Unassigned	5108	1522	4381	1148	51679
All	Unclassifiable	145	89	211	80	2634
All	Total	17714	4387	11286	3138	135320
Cytomorphology	Treated	0	338	234	224	1335
Cytomorphology	AML	1545	198	335	102	849
Cytomorphology	MDS	1533	338	700	199	943
Cytomorphology	MDS/MPN	453	212	381	151	470
Cytomorphology	MPN	297	55	1003	133	3374
Cytomorphology	Lymphoid	136	43	138	31	6901
Cytomorphology	MM	71	29	90	13	3645
Cytomorphology	Non/Pre-HM	4626	714	2620	393	31977
Cytomorphology	Other HM	94	11	33	9	230
Cytomorphology	Unassigned	149	67	167	52	1088
Cytomorphology	Unclassifiable	72	8	64	13	603
Cytomorphology	Total	8976	2013	5765	1320	51415
Immunophenotyping	Treated	0	313	209	200	1341
Immunophenotyping	AML	1431	272	346	146	805
Immunophenotyping	MDS	1339	344	707	201	916
Immunophenotyping	MDS/MPN	383	186	335	131	377
Immunophenotyping	MPN	244	47	797	100	2616
Immunophenotyping	Lymphoid	141	68	193	50	13499
Immunophenotyping	MM	56	33	89	10	3617
Immunophenotyping	Non/Pre-HM	4013	821	2822	469	42998
Immunophenotyping	Other HM	72	15	34	13	328
Immunophenotyping	Unassigned	96	44	120	39	810
Immunophenotyping	Unclassifiable	62	87	198	76	2457
Immunophenotyping	Total	7837	2230	5850	1435	69764
Cytogenetics	Treated	0	137	176	121	391
Cytogenetics	AML	1741	182	315	96	687
Cytogenetics	MDS	1602	300	611	162	747
Cytogenetics	MDS/MPN	454	154	238	87	213

		ID	ID-FU	FU	FU-FU	No-UBA1
Cytogenetics	MPN	322	44	643	73	1431
Cytogenetics	Lymphoid	150	34	84	17	4485
Cytogenetics	MM	71	21	67	8	509
Cytogenetics	Non/Pre-HM	4939	557	1154	246	6314
Cytogenetics	Other HM	103	10	29	6	156
Cytogenetics	Unassigned	2049	641	1160	365	9402
Cytogenetics	Unclassifiable	87	4	52	7	238
Cytogenetics	Total	11518	2084	4529	1188	24573
FISH	Treated	0	107	152	115	585
FISH	AML	1686	127	297	76	583
FISH	MDS	1306	235	499	121	550
FISH	MDS/MPN	322	88	155	49	146
FISH	MPN	172	19	303	45	701
FISH	Lymphoid	116	34	114	14	7716
FISH	MM	70	35	97	11	4095
FISH	Non/Pre-HM	2768	291	648	114	6917
FISH	Other HM	96	2	26	5	105
FISH	Unassigned	1580	503	975	295	16130
FISH	Unclassifiable	66	5	47	4	312
FISH	Total	8182	1446	3313	849	37840

ID: Patients referred to at initial diagnosis. ID-FU: Patients whose first sample was referred to as initial diagnosis and had further follow-up. FU: Patients referred to as follow-up. FU-FU: Patients whose first sample was referred to as follow-up and had further follow up. No-UBA1: Patients who did not undergo *UBA1*-including myeloid gene panel. NR: Data not retrieved. BM: Bone marrow. PB: Peripheral blood. Other: Samples other than BM or PB or those with multiple sample types. Treated: Patients whose samples were referred for treatment response. AML: Acute myeloid leukemia. MDS: Myelodysplastic neoplasms. MDS/MPN: Myelodysplastic/myeloproliferative neoplasms. MPN: Myeloproliferative neoplasms. Lymphoid: Non-myeloid malignancies except multiple myeloma. MM: multiple myeloma. Non/Pre-HM: premalignant or non-diagnostic cases. Other HM: Other myeloid or mixed myeloid and lymphoid malignancies. Unassigned: Patients with molecular genetic examination only. Unclassifiable: Unclear diagnosis without clear myeloid or lymphoid specification and samples without diagnostic power due to low sample quality.

Supplementary Table 2

Myeloid.panel.genes		
ASXL1	*GNB1*	*RAD21*
ASXL2	*IDH1*	*RUNX1*
ATRX	*IDH2*	*SETBP1*
BCOR	*IL6R*	*SF1*
BCORL1	*JAK2*	*SF3A1*
BRAF	*KIT*	*SF3B1*
CALR	*KRAS*	*SH2B3*
CBL	*MPL*	*SMC1A*
CEBPA	*MYD88*	*SMC3*
CSF3R	*NF1*	*SRSF2*
CSNK1A1	*NOTCH1*	*STAG2*
CUX1	*NPM1*	*SUZ12*
DDX41	*NRAS*	*TET2*
DNMT3A	*PDGFRA*	*TP53*
ETNK1	*PDGFRB*	*U2AF1*
ETV6	*PHF6*	*U2AF2*
EZH2	*PIGA*	*UBA1*
FBXW7	*PPM1D*	*WT1*
FLT3	*PRPF8*	*ZEB2*
GATA1	*PTEN*	*ZRSR2*
GATA2	*PTPN11*	

Supplementary Table 3

Variant	Frequency
M41T	69
M41L	34
M41V	33
M41ss	16
S56F	15
A478S	14
K746N/E/I/del	8
D506N/G	6
S621C	6
D585E	6
S56Y	4
I890M/F	4
I301V	4
Y618C	4
L59/R	4
I894S/F	3
N139H/K/Y	3
N606I	3
R869L	2
G95W	2
I50T/K	2
E1049K	2
S620P	2

Supplementary Table 4

ID	Age	Sex	Class	Variant	DNA	VAF	Tier	Mat	IDFU	Diagnosis	Comutation	Karytotype	comment
1	54	F	M41	M41V	c.121A>G	50,7	Tier-1	BM	ID	non_preHM	*TP53*	45,X,-X[20]/47,XX,+X[2]	
2	67	M	M41	M41L	c.121A>C	81,4	Tier-1	BM	ID	non_preHM		46,XY[20]	
3	69,8	M	M41	M41L	c.121A>C	79,2	Tier-1	BM	ID	non_preHM		46,XY[20]	
4	75	M	M41	M41L	c.121A>C	78,6	Tier-1	BM	FU	MDS	*DNMT3A*	no metaphases	
5	85	F	M41	M41T	c.122T>C	3,7	Tier-1	BM	FU	MDS	*EZH2,RUNX1,SF3B1,TET2*	45,X,-X,i(3)(q10)[4]/46,XX[16]	
6	81	M	M41	M41T	c.122T>C	45,3	Tier-1	PB	ID	unassigned			
7	73	M	M41	M41T	c.122T>C	89,2	Tier-1	PB	FU	unassigned	*DNMT3A*		
8	71	M	M41	SS	c.118-1G>C	62,3	Tier-1	BM	ID	MM		no metaphases	
9	82	M	M41	SS	c.118-1G>C	57,5	Tier-1	BM	ID	unassigned	*TET2*	45,X,-Y[11]/46,XY[9]	
10	64	M	M41	M41L	c.121A>C	60,9	Tier-1	PB	FU	MDS		46,XY[11]	
11	69	M	M41	M41V	c.121A>G	4,58	Tier-1	BM	ID	MDS		46,XY[20]	AML-Prog
12	77	M	M41	M41T	c.122T>C	89,5	Tier-1	BM	FU	unassigned			vexas-sus
13	77	M	M41	M41V	c.121A>G	23,6	Tier-1	PB	ID	unassigned	*DNMT3A*		
14	69	M	M41	SS	c.118-1G>C	67,4	Tier-1	BM	FU	MDS		46,XY[20]	
15	78	M	M41	M41T	c.122T>C	69,3	Tier-1	PB	ID	non_preHM			
16	77	M	M41	M41L:M41V	c.121A>C:c.121A>G	40.3:31.3	Tier-1, Tier-1	PB	FU	unassigned			
17	72	M	M41	M41T	c.122T>C	56,2	Tier-1	PB	ID	unassigned			
18	80	M	M41	M41T	c.122T>C	75,6	Tier-1	BM	FU	unassigned		46,XY[24]	vexas-sus
19	70	F	M41	M41V	c.121A>G	45,1	Tier-1	PB	ID	non_preHM			
20	82	M	M41	M41L:K43R	c.121A>C:c.128A>G	71.8:70.5	Tier-1, Tier-3	BM	ID	non_preHM	*DNMT3A*	46,XY[11]	
21	73	M	M41	M41L	c.121A>C	80	Tier-1	BM	FU	non_preHM		46,XY[22]	
22	80	M	M41	M41T	c.122T>C	65,8	Tier-1	BM	FU	MDS		46,XY[20]	
23	82	M	M41	M41L	c.121A>C	34,7	Tier-1	BM	FU	MDS	*SF3B1*	46,XY[6]	
24	75	M	M41	M41T	c.122T>C	40,2	Tier-1	BM	ID	non_preHM	*ASXL1*	46,XY[20]	
25	72	M	M41	M41L	c.121A>C	80	Tier-1	BM	FU	non_preHM		46,XY[15]	
26	63	M	M41	M41L	c.121A>C	3,33	Tier-1	PB	ID	unassigned	*SF3B1,SRSF2,TET2,ZRSR2*		
27	67	M	M41	M41L	c.121A>C	56	Tier-1	PB	FU	unassigned			
28	82	M	M41	M41V	c.121A>G	72,1	Tier-1	BM	ID	non_preHM		46,XY[20]	
29	84	M	M41	M41T	c.122T>C	54,4	Tier-1	BM	ID	non_preHM	*SRSF2*	46,XY[20]	
30	85	M	M41	M41T	c.122T>C	2,65	Tier-1	BM	ID	non_preHM	*ETV6,U2AF1*	46,XY,del(20)(q11q13)[8]/46,XY[13]	
31	68	M	M41	SS	c.118-1G>C	21,5	Tier-1	BM	FU	unassigned	*U2AF1*	46,XY[17]	
32	78	M	M41	M41V	c.121A>G	75,2	Tier-1	BM	FU	non_preHM		46,XY[20]	
33	73	M	M41	M41L	c.121A>C	3,91	Tier-1	BM	FU	MDS	*DNMT3A,EZH2*	46,XY[20]	vexas-sus
34	64	M	M41	M41T	c.122T>C	82,2	Tier-1	BM	FU	non_preHM	*EZH2*	46,XY,del(2)(p25p23)[7]/46,XY[13]	
35	83	M	M41	SS:SS	c.118-2delinsCC:c.118-1G>C	4.1:83	Tier-1, Tier-1	BM	ID	unassigned		45,X,-Y[14]/46,XY[6]	
36	67	M	M41	M41T	c.122T>C	75,8	Tier-1	PB	FU	non_preHM	*DNMT3A,TET2*		
37	74	M	M41	M41L	c.121A>C	73,3	Tier-1	BM	ID	non_preHM		46,XY[20]	
38	67	M	M41	M41V	c.121A>G	74,6	Tier-1	BM	FU	unassigned	*U2AF1*	46,XY[20]	
39	74	M	M41	M41T	c.122T>C	78,7	Tier-1	BM	ID	non_preHM		46,XY[20]	
40	79	M	M41	M41V	c.121A>G	27,1	Tier-1	BM	ID	non_preHM		45,X,-Y[20]/46,XY[4]	
41	79	M	M41	M41T	c.122T>C	4,7	Tier-1	BM	ID	non_preHM	*DNMT3A,TET2*	46,XY[20]	
42	84	M	M41	M41T	c.122T>C	18,1	Tier-1	BM	FU	non_preHM		46,XY[20]	
43	73	M	M41	M41T	c.122T>C	78,2	Tier-1	BM	FU	unassigned		45,X,-Y[20]/46,XY[2]	
44	88	M	M41	M41L	c.121A>C	42,7	Tier-1	PB	ID	non_preHM			
45	71	M	M41	M41L	c.121A>C	71,8	Tier-1	BM	FU	non_preHM		46,XY[22]	
46	81	M	M41	M41T	c.122T>C	33,2	Tier-1	BM	ID	non_preHM		46,XY,del(13)(q14q33)[7]/46,XY[13]	
47	76	M	M41	M41T	c.122T>C	10,2	Tier-1	PB, BM	ID	unassigned	*DNMT3A,TET2*	46,XY[20]	
48	70	M	M41	M41V	c.121A>G	88	Tier-1	PB	ID	unassigned			vexas-sus
49	64	M	M41	M41V	c.121A>G	35,6	Tier-1	PB	ID	unassigned			vexas-sus
50	61	M	M41	M41L	c.121A>C	88,4	Tier-1	BM	FU	non_preHM			vexas-sus
51	76	M	M41	M41T	c.122T>C	68,7	Tier-1	BM	ID	MDS	*EZH2*	47,XY,+8[8]/46,XY[12]	
52	79	M	M41	M41V	c.121A>G	79	Tier-1	BM	FU	MDS		46,XY[23]	

ID	Age	Sex	Class	Variant	DNA	VAF	Tier	Mat	IDFU	Diagnosis	Comutation	Karytotype	comment
53	83	M	M41	M41V	c.121A>G	50,1	Tier-1	BM	ID	non_preHM	*NF1,TET2*	46,XY.ish del(13)(q14q14)(DLEU-)[19]/45,X,-Y[13]/46,XY[2]	
54	79	M	M41	M41T	c.122T>C	74,3	Tier-1	BM	FU	non_preHM		46,XY[20]	
55	64	M	M41	M41T	c.122T>C	88,4	Tier-1	BM	ID	unassigned		46,XY[21]	vexas-sus
56	76	M	M41	M41L	c.121A>C	62	Tier-1	PB	ID	unassigned			vexas-sus
57	76	F	M41	M41V	c.121A>G	5	Tier-1	PB	FU	unassigned	*SF3B1*		
58	89	M	M41	M41T	c.122T>C	47	Tier-1	BM	ID	non_preHM		46,XY[21]	
59	83	M	M41	M41T	c.122T>C	76,5	Tier-1	BM	FU	non_preHM		45,X,-Y[19]/46,XY[1]	
60	65	M	M41	M41T	c.122T>C	56,2	Tier-1	BM	FU	non_preHM		46,XY[20]	vexas-sus
61	78	M	M41	M41L	c.121A>C	84,1	Tier-1	BM	FU	MDS		46,XY[20]	
62	67	M	M41	M41V	c.121A>G	93	Tier-1	BM	FU	unassigned	*DNMT3A*	no metaphases	
63	64	M	M41	M41V	c.121A>G	95,1	Tier-1	BM	ID	MDS	*PPM1D*	46,XY[21]	vexas-sus
64	82	M	M41	M41T	c.122T>C	20,8	Tier-1	PB	ID	unassigned	*SH2B3*		
65	61	M	M41	SS	c.118-1G>C	82,6	Tier-1	BM	ID	MDS		45,X,-Y[16]/46,XY[4]	
66	65	M	M41	M41L	c.121A>C	66,8	Tier-1	PB	ID	unassigned			vexas-sus
67	87	M	M41	M41T	c.122T>C	59,4	Tier-1	BM	ID	non_preHM		46,XY[20]	
68	78	M	M41	M41V	c.121A>G	58,5	Tier-1	BM	ID	unassigned			vexas-sus
69	83	M	M41	M41T	c.122T>C	3,54	Tier-1	BM	ID	non_preHM		45,X,-Y[9]/46,XY[11]	
70	83	M	M41	M41L	c.121A>C	35,6	Tier-1	BM	FU	MDS		46,XY[21]	
71	78	M	M41	M41T	c.122T>C	73,7	Tier-1	BM	ID	unassigned	*TET2*	46,XY[20]	
72	90	M	M41	M41T	c.122T>C	30,2	Tier-1	BM	ID	MDS	*RAD21,SF3B1,TET2*	46,XY[10]	
73	56	M	M41	M41T	c.122T>C	62	Tier-1	BM	FU	unassigned	*ZRSR2*	46,XY[20]	vexas-sus
74	81	M	M41	M41T	c.122T>C	96,2	Tier-1	BM	ID	non_preHM	*DNMT3A*	no metaphases	
75	77	M	M41	M41L	c.121A>C	69,1	Tier-1	BM	FU	non_preHM	*DNMT3A*	46,XY[20]	
76	79	M	M41	M41V	c.121A>G	60	Tier-1	PB	FU	unassigned	*TET2*		
77	83	M	M41	M41T	c.122T>C	75,8	Tier-1	BM	ID	non_preHM	*ASXL1,DNMT3A*	46,XY[20]	
78	83	M	M41	SS	c.118-1G>C	16,6	Tier-1	BM	ID	MDS	*JAK2*	45,X,-Y[19]/46,XY[1]	
79	81	M	M41	SS	c.118-1G>C	80,3	Tier-1	BM	ID	non_preHM		45,X,-Y[6]/46,XY[14]	
80	58	M	M41	M41T	c.122T>C	69,4	Tier-1	BM	ID	non_preHM		46,XY[20]	
81	81	M	M41	M41T	c.122T>C	14,8	Tier-1	PB	ID	unassigned			
82	74	M	M41	M41L	c.121A>C	82,7	Tier-1	BM	ID	unassigned		46,XY[20]	
83	83	M	M41	M41T	c.122T>C	2,38	Tier-1	BM	ID	MM	*DNMT3A,SRSF2*	46,XY[20]	
84	61	M	M41	M41T	c.122T>C	73,6	Tier-1	BM	ID	non_preHM		46,XY[20]	
85	64	M	M41	M41V	c.121A>G	31,4	Tier-1	BM	ID	non_preHM		46,XY[20]	
86	68	M	M41	SS	c.118-1G>C	96,2	Tier-1	BM	FU	MDS		no metaphases	
87	68	M	M41	M41L	c.121A>C	70,5	Tier-1	PB, BM	ID	MDS			vexas-sus
88	65	M	M41	M41T	c.122T>C	83,5	Tier-1	BM	ID	non_preHM		46,XY[20]	
89	85	M	M41	M41T	c.122T>C	89,9	Tier-1	BM	ID	non_preHM	*DNMT3A*	46,XY[20]	
90	59	M	M41	M41L	c.121A>C	38,6	Tier-1	PB	FU	unassigned			
91	76	M	M41	M41T	c.122T>C	36,8	Tier-1	PB	FU	unassigned			vexas-sus
92	87	M	M41	M41T	c.122T>C	4,45	Tier-1	PB	ID	non_preHM	*SMC1A*		
93	76	M	M41	M41V	c.121A>G	35	Tier-1	BM	FU	non_preHM			
94	46	M	M41	M41T	c.122T>C	73,1	Tier-1	PB	ID	unassigned			vexas-sus
95	52	M	M41	M41L	c.121A>C	82,8	Tier-1	BM	ID	non_preHM			vexas-sus
96	70	M	M41	M41T	c.122T>C	51,8	Tier-1	BM	ID	unassigned		46,XY[20]	vexas-sus
97	71	M	M41	M41V	c.121A>G	79,2	Tier-1	BM	FU	MDS		46,XY[11]	vexas-sus
98	72	M	M41	M41T	c.122T>C	78,9	Tier-1	BM	ID	non_preHM		46,XY[20]	
99	72	M	M41	M41L	c.121A>C	56,9	Tier-1	BM	ID	non_preHM	*DNMT3A,TET2*	46,XY[20]	
100	79	M	M41	M41T	c.122T>C	73,8	Tier-1	BM	ID	non_preHM		45,X,-Y[19]/46,XY[1]	
101	67	M	M41	M41L	c.121A>C	9	Tier-1	PB	ID	unassigned			vexas-sus
102	61	M	M41	M41T	c.122T>C	49,8	Tier-1	PB, BM	ID	non_preHM	*PRPF8*		
103	72	M	M41	M41V	c.121A>G	33,7	Tier-1	PB, BM	ID	unassigned			vexas-sus
104	76	M	M41	M41V	c.121A>G	50,5	Tier-1	BM	FU	MPN	*CBL,DNMT3A,JAK2,MPL*	48,XY,+8,+9[9]/46,XY[11]	
105	72	M	M41	M41T	c.122T>C	76,6	Tier-1	PB	ID	unassigned	*DNMT3A*		
106	74	M	M41	SS	c.118-1G>C	80,6	Tier-1	BM	ID	non_preHM		46,XY[21]	
107	69	M	M41	M41L	c.121A>C	85,1	Tier-1	BM	ID	MDS		46,XY[23]	
108	83	M	M41	SS	c.118-1G>C	56,1	Tier-1	PB	FU	non_preHM	*DNMT3A*	no metaphases	

ID	Age	Sex	Class	Variant	DNA	VAF	Tier	Mat	IDFU	Diagnosis	Comutation	Karytotype	comment
109	71	M	M41	M41T	c.122T>C	70,1	Tier-1	BM	ID	non_preHM	*DNMT3A*	46,XY[20]	
110	70	M	M41	M41V	c.121A>G	50,1	Tier-1	BM	ID	non_preHM	*ZRSR2*	46,XY[20]	
111	69	M	M41	M41T	c.122T>C	76,4	Tier-1	BM	ID	non_preHM		46,XY[20]	
112	83	M	M41	M41T	c.122T>C	79,2	Tier-1	BM	ID	MDS	*CBL,EZH2,U2AF1*	46,XY[20]	
113	67	M	M41	M41T	c.122T>C	66,7	Tier-1	BM	ID	unassigned		46,XY[20]	vexas-sus
114	70	M	M41	M41L	c.121A>C	75,2	Tier-1	BM	ID	non_preHM		46,XY[30]	
115	83	M	M41	M41T	c.122T>C	75,1	Tier-1	BM	FU	non_preHM		46,XY[20]	
116	85	M	M41	M41T	c.122T>C	72,6	Tier-1	BM	FU	non_preHM		45,X,-Y[10]/46,XY[10]	
117	71	M	M41	M41T	c.122T>C	90	Tier-1	BM	FU	unassigned		46,XY[20]	vexas-sus
118	86	M	M41	M41V	c.121A>G	59,7	Tier-1	BM	FU	Lymphoid	*MYD88*	45,X,-Y[23]/46,XY[7]	
119	61	F	M41	M41T	c.122T>C	71,8	Tier-1	PB	ID	unassigned			vexas-sus
120	73	M	M41	M41T	c.122T>C	90,6	Tier-1	BM	FU	MDS		46,XY[20]	
121	73	M	M41	M41T:S56F	c.122T>C:c.167C>T	14.2:61.7	Tier-1, Tier-1	BM	ID	non_preHM	*DNMT3A*	46,XY[20]	
122	71	M	M41	M41T:G261R	c.122T>C:c.781G>A	62.7:100	Tier-1, Tier-3	BM	ID	non_preHM		46,XY[20]	
123	69	M	M41	SS	c.118-1G>C	29	Tier-1	PB	ID	MDS	*CBL,CUX1,JAK2,SRSF2,TET2*		
124	77	M	M41	M41V	c.121A>G	28,5	Tier-1	BM	FU	MDS	*DNMT3A*	45,X,-Y[8]/46,XY[12]	
125	63	M	M41	M41T	c.122T>C	37,5	Tier-1	BM	ID	unassigned	*TET2*	45,X,-Y[20]/45,X,-Y,der(9)t(9;15)(p24;q12)[2]	
126	70	M	M41	M41L	c.121A>C	81,8	Tier-1	BM	FU	unassigned		46,XY[20]	
127	62	M	M41	SS	c.118-1G>C	86,1	Tier-1	BM	ID	unassigned	*DNMT3A*	46,XY[20]	
128	76	M	M41	L59Q:M41V	c.176T>A:c.121A>G	2.94:15.8	Tier-2, Tier-1	PB	FU	MDS	*SF3B1*	no metaphases	
129	85	M	M41	M41T	c.122T>C	63,8	Tier-1	BM	ID	MDS		45,X,-Y[15]/46,XY[5]	
130	61	M	M41	M41T	c.122T>C	31,1	Tier-1	BM	FU	non_preHM		46,XY[20]	vexas-sus
131	68	M	M41	M41L	c.121A>C	51,3	Tier-1	BM	FU	non_preHM	*DNMT3A*	46,XY[20]	
132	74	M	M41	M41T	c.122T>C	75	Tier-1	BM	ID	unassigned		46,XY[20]	
133	74	M	M41	M41V	c.121A>G	56	Tier-1	PB	ID	unassigned			
134	82	M	M41	M41L	c.121A>C	3,09	Tier-1	BM	ID	MDS	*SF3B1,TET2,ZRSR2*	47,XY,+8[2]/46,XY[18]	
135	76	M	M41	M41V:E1049K	c.121A>G:c.3145G>A	48.2:15.5	Tier-1, Tier-2	BM	ID	unassigned			vexas-sus
136	84	M	M41	M41V	c.121A>G	64,4	Tier-1	BM	ID	unassigned	*DNMT3A,RAD21,TET2*	46,XY[22]	
137	76	M	M41	M41T	c.122T>C	76,6	Tier-1	PB	FU	unassigned			vexas-sus
138	67	M	M41	M41V:nonM41SS	c.121A>G:c.346-2A>G	49:03,2	Tier-1, Tier-2	PB	ID	unassigned			
139	76	M	M41	SS	c.118-5_118-1del	45,4	Tier-1	BM	ID	non_preHM	*DNMT3A,TET2*	46,XY[20]	
140	90	F	M41	SS	c.118-2A>G	17,6	Tier-1	BM	ID	unassigned	*SRSF2*	46,XX[22]	
141	68	M	M41	M41L	c.121A>C	44,1	Tier-1	BM	FU	non_preHM		46,XY[21]	
142	69	M	M41	M41T	c.122T>C	72,4	Tier-1	PB	FU	unassigned			vexas-sus
143	66	M	M41	M41T	c.122T>C	32,2	Tier-1	BM	ID	non_preHM	*IDH2*	46,XY[20]	vexas-sus
144	76	M	M41	M41T	c.122T>C	47,7	Tier-1	BM	ID	non_preHM		45,X,-Y[18]/46,XY[2]	
145	61	M	M41	M41T	c.122T>C	82	Tier-1	BM	ID	unassigned		46,XY[20]	
146	75	M	M41	M41L	c.121A>C	86,7	Tier-1	BM	FU	non_preHM		46,XY[20]	vexas-sus
147	72	M	M41	M41V	c.121A>G	25,6	Tier-1	BM	ID	unassigned		46,XY[20]	
148	82	M	M41	M41T	c.122T>C	21,6	Tier-1	BM	ID	non_preHM		46,XY[20]	
149	62	M	M41	SS	c.118-1G>C	80,2	Tier-1	BM	FU	non_preHM		46,XY[20]	
150	72	M	M41	M41V	c.121A>G	26,5	Tier-1	BM	ID	unassigned	*TP53,U2AF1*	46,XY[20]	
151	64	M	M41	M41T	c.122T>C	70,5	Tier-1	BM	FU	MDS	*DNMT3A*	46,XY[20]	
152	73	M	recur	D506N:R869L	c.1516G>A:c.2606G>T	4.75:4.38	Tier-1, Tier-2	BM	ID	AML	*ASXL1,FLT3,KRAS,NRAS,SETBP1,SRSF2*	46,XY,i(17)(q10)[2]/46,XY[5]	
153	76	M	recur	S56F	c.167C>T	2,55	Tier-1	BM	ID	unassigned	*SRSF2*	46,XY[5]	
154	70	M	recur	S56F:I301V:I890F	c.167C>T:c.901A>G:c.2668A>T	29.9:14.3:10.8	Tier-1, Tier-2, Tier-2	BM	FU	MDS		46,XY[23]	hyper-Bil
155	61	M	recur	S621C	c.1861A>T	78	Tier-1	BM	FU	unassigned		46,XY[20]	hemolysis
156	78	M	recur	A478S	c.1432G>T	59,7	Tier-1	PB	ID	MDS/MPN	*ASXL1*		
157	69	M	recur	S621C	c.1861A>T	76	Tier-1	BM	FU	MDS	*IDH2,JAK2,TET2,ZRSR2*	46,XY[20]	hemolysis
158	54	M	recur	S56F:L59R:I890F	c.167C>T:c.176T>G:c.2668A>T	11.7:7.9:14.3	Tier-1, Tier-2, Tier-2	BM	ID	unassigned			
159	80	M	recur	D506G:K746N:K746N	c.1517A>G:c.2238A>C:c.2238A>T	58:23.7:35.3	Tier-1, Tier-2, Tier-2	BM	FU	MDS	*EZH2,SF3B1,TET2*	47,XY,+8[14]/46,XY[6]	
160	69	M	recur	S621C	c.1861A>T	52,9	Tier-1	BM	ID	non_preHM		46,XY[20]	
161	82	M	recur	D506N	c.1516G>A	24,5	Tier-1	BM	ID	MDS	*ASXL1,CBL,EZH2,KRAS,PHF6,TET2*	46,XY[30]	

ID	Age	Sex	Class	Variant	DNA	VAF	Tier	Mat	IDFU	Diagnosis	Comutation	Karytotype	comment
162	64	M	recur	A478S	c.1432G>T	2,82	Tier-1	BM	ID	AML	*ASXL1,DNMT3A,KRAS,NRAS,PPM1D,U2AF1*	45,XY,der(3)t(3;21)(q26;q22),der(7;8)(q10;q10),der(17)del(17)(p13p12)t(3;17)(q26;q25),der(21)t(17;21)(q25;q22)[11]/46,XY,del(11)(q14q25)[2]/46,XY[11]	
163	80	M	recur	S56F	c.167C>T	72,5	Tier-1	BM	FU	non_preHM	*NF1,SRSF2,TET2*	46,XY[18]	CMML-Prog
164	71	M	recur	A478S	c.1432G>T	93,7	Tier-1	BM	FU	MDS	*ETV6,SETBP1,SRSF2*	46,XY[20]	
165	58	M	recur	S56F:R869L:I894F	c.167C>T:c.2606G>T:c.2680A>T	53.9:2.31:13.2	Tier-1, Tier-2, Tier-2	BM	ID	Lymphoid		46,XY[20]	hemolysis-sus
166	70	M	recur	D506N	c.1516G>A	35	Tier-1	BM	ID	non_preHM	*DNMT3A,U2AF1*	46,XY[20]	
167	70	M	recur	S56Y:S56F	c.167C>A:c.167C>T	63.3:21.2	Tier-2, Tier-1	BM	ID	MDS		46,XY[20]	hemolysis-sus
168	78	M	recur	S621C	c.1861A>T	17,5	Tier-1	PB	ID	non_preHM	*BCORL1,TET2,U2AF1*		
169	73	M	recur	S56F	c.167C>T	78,6	Tier-1	BM	ID	MDS	*KRAS*	46,XY[14]	
170	73	M	recur	A478S	c.1432G>T	81,2	Tier-1	PB	ID	unassigned	*ASXL1,CBL,EZH2,TET2*		
171	81	M	recur	S56F	c.167C>T	49,4	Tier-1	BM	ID	non_preHM	*DNMT3A,SF3B1*	46,XY[21]	
172	68	M	recur	A478S	c.1432G>T	88	Tier-1	BM	FU	unassigned	*CSF3R,PRPF8,U2AF1*	46,XY,r(7)(p13q11)[4]/45,XY,-7[7]/46,XY[2]	
173	54	M	recur	A478S	c.1432G>T	88,3	Tier-1	BM	ID	MDS/MPN	*DNMT3A,RUNX1,TET2*	46,XY[22]	hemolysis
174	80	M	recur	S56F	c.167C>T	13,5	Tier-1	PB, BM	ID	MDS	*TET2*		
175	82	M	recur	S621C	c.1861A>T	2,82	Tier-1	BM	ID	AML	*IDH1,IDH2,TP53*	43,XY,der(2)t(2;5)(q24;q34),der(5)t(2;5)(q24;q13),dic(7;20)(q22;q11),dic(14;17)(p11;q25),-18[6]/46,XY,der(2)t(2;5)(q24;q34),der(5)t(2;5)(q24;q13),dic(7;20)(q22;q11),+8,dic(14;17)(p11;q25),-18,+der(20)(::20p12->20q11::7q22->7q31::)x2[2]/46,XY[7]	
176	76	M	recur	A478S	c.1432G>T	79,4	Tier-1	BM	ID	MDS	*CBL*	46,XY[20]	
177	70	M	recur	D506N	c.1516G>A	36	Tier-1	PB	ID	unassigned			vexas-sus
178	67	M	recur	A478S	c.1432G>T	91,1	Tier-1	BM	ID	non_preHM	*IDH2,TET2*	46,XY[20]	CMML-Prog, hyper-Bil
179	61	M	recur	A478S	c.1432G>T	91,3	Tier-1	BM	ID	unassigned			hemolysis
180	64	M	recur	S56F	c.167C>T	73,6	Tier-1	BM	FU	unassigned	*NF1,PTPN11*		
181	71	M	recur	D506G	c.1517A>G	9	Tier-1	PB	FU	MPN	*ASXL1,JAK2,SRSF2*		
182	75	M	recur	S56F	c.167C>T	56	Tier-1	BM	ID	MDS		46,XY[20]	hyper-Bil
183	79	M	recur	A478S	c.1432G>T	40,8	Tier-1	BM	FU	MDS	*NOTCH1,SRSF2,TET2*	46,XY[20]	
184	62	M	recur	S56F	c.167C>T	42,6	Tier-1	BM	ID	unassigned			
185	71	M	recur	A478S	c.1432G>T	88,5	Tier-1	BM	ID	unassigned	*ASXL1*	46,XY[21]	
186	77	M	recur	A478S	c.1432G>T	4,25	Tier-1	BM	ID	unassigned	*CBL,IDH2,KIT,SMC1A,SRSF2,TET2*	46,XY[21]	
187	55	M	recur	S621C	c.1861A>T	82	Tier-1	BM	FU	non_preHM		no metaphases	
188	67	M	recur	S56F	c.167C>T	63,4	Tier-1	BM	FU	non_preHM		46,XY[20]	hyper-Bil
189	86	M	recur	A478S	c.1432G>T	40,4	Tier-1	PB	ID	non_preHM	*IDH1,KRAS,SRSF2*		
190	79	M	recur	A478S	c.1432G>T	67,3	Tier-1	BM	FU	MDS	*ASXL1,TET2*	46,XY[20]	
191	78	M	recur	S56F	c.167C>T	72	Tier-1	BM	FU	MDS	*JAK2,SETBP1*	46,XY[13]	
192	76	M	recur	I890M	c.2670C>G	84,3	Tier-3	BM	ID	AML	*NPM1,TET2*	46,XY[16]	
193	72	M	recur	I301V	c.901A>G	68,9	Tier-2	BM	FU	unassigned			
194	80	M	recur	I50T	c.149T>C	2,87	Tier-2	BM	ID	non_preHM	*NRAS*	45,X,-Y[19]/46,XY[1]	
195	85	M	recur	G95W	c.283G>T	73	Tier-2	PB	ID	MDS/MPN			
196	57	M	recur	S620P	c.1858T>C	64,4	Tier-3	BM	ID	non_preHM		46,XY[20]	
197	62	M	recur	N139Y	c.415A>T	13,9	Tier-2	PB	ID	unassigned	*ASXL1*		
198	54	M	recur	L59Q	c.176T>A	43,3	Tier-2	BM	ID	MDS	*SRSF2*	46,XY[20]	vexas-sus
199	64	M	recur	I890F	c.2668A>T	79,3	Tier-2	BM	FU	MPN	*PHF6*	46,XY[14]	
200	65	M	recur	G95W	c.283G>T	90,5	Tier-2	PB	FU	MDS/MPN	*NF1,NRAS*		AML-Prog
201	70	M	recur	I301V	c.901A>G	95,3	Tier-2	BM	FU	unassigned			hemolysis
202	63	M	recur	D585E	c.1755C>A	3,93	Tier-2	BM	ID	non_preHM	*ASXL1,CBL,IDH2,SETBP1,SRSF2*	46,XY[20]	hyper-Bil
203	76	M	recur	D585E	c.1755C>G	18	Tier-2	BM	ID	MDS	*DNMT3A,EZH2*	45,XY,der(21;22)(q10;q10)[15]/45,XY,del(7)(q21q34),der(21;22)(q10;q10)[7]/45,XY,del(7)(q21q36),del(20)(q11q13),der(21;22)(q10;q10)[5]	
204	69	M	recur	I50K	c.149T>A	4,17	Tier-2	BM	ID	unassigned		46,XY[20]	
205	67	M	recur	D585E	c.1755C>G	55	Tier-2	BM	FU	unassigned	*ASXL1*	46,XY[20]	
206	73	M	recur	D585E	c.1755C>G	10,9	Tier-2	BM	FU	MPN	*ASXL1,JAK2*	46,XY[10]	

ID	Age	Sex	Class	Variant	DNA	VAF	Tier	Mat	IDFU	Diagnosis	Comutation	Karytotype	comment
207	64	M	recur	N606I	c.1817A>T	55,5	Tier-2	BM	FU	AML	*ASXL1,CSF3R,NRAS,RUNX1,SETBP1,SRSF2,STAG2*	47,XY,+21[14]/46,XY[6]	AML-Prog
208	72	M	recur	N139K	c.417C>G	92	Tier-2	BM	ID	MDS/MPN	*ASXL1,CBL,KRAS,PTPN11*	46,XY[20]	
209	67	M	recur	K746E	c.2236A>G	8,5	Tier-2	BM	FU	unassigned	*SETBP1,U2AF1*		
210	69	M	recur	Y618C	c.1853A>G	97,7	Tier-2	BM	ID	MDS/MPN	*ASXL1,CBL,SRSF2*	46,XY[20]	
211	67	M	recur	K746E	c.2236A>G	33,9	Tier-2	BM	FU	unassigned	*ASXL1,SRSF2,TET2*		AML-Prog
212	77	M	recur	L59Q	c.176T>A	7,8	Tier-2	BM	FU	unassigned		45,X,-Y[17]/46,XY[3]	
213	71	M	recur	S56Y	c.167C>A	25,7	Tier-2	BM	ID	non_preHM		46,XY,der(7)(::7p15->7q11::7q31->7q32::)[3]/45,XY,-7[3]/46,XY[14]	
214	77	M	recur	E1049K	c.3145G>A	94,4	Tier-2	BM	ID	non_preHM	*RUNX1,SRSF2,TET2*	46,XY[20]	hyper-Bil
215	58	M	recur	Y618C	c.1853A>G	20,2	Tier-2	BM	ID	MDS	*GATA2,RUNX1*	46,XY[30]	
216	80	M	recur	D585E	c.1755C>G	8,2	Tier-2	PB	ID	non_preHM	*DNMT3A*		
217	82	M	recur	S56Y	c.167C>A	68,8	Tier-2	PB	FU	MDS	*TET2*		
218	63	M	recur	K746E	c.2236A>G	82,9	Tier-2	BM	ID	MDS	*SRSF2,TET2*	46,XY[20]	
219	72	M	recur	K746E	c.2236A>G	76,2	Tier-2	PB	FU	AML	*KRAS*	46,XY,del(5)(q21q34)[22]	AML-Prog, hyper-Bil
220	74	M	recur	Y618C	c.1853A>G	2,26	Tier-2	BM	FU	MPN	*ASXL1,JAK2*	46,XY[20]	
221	77	M	recur	I894F	c.2680A>T	86,9	Tier-2	BM	FU	non_preHM		46,XY[20]	hyper-Bil
222	59	M	recur	S620P	c.1858T>C	42,5	Tier-3	PB	ID	non_preHM	*ASXL1,RUNX1,SETBP1,SRSF2*	46,XY[26]	
223	69	M	recur	N606I	c.1817A>T	33,2	Tier-2	PB, BM	ID	MDS			vexas-sus
224	71	M	recur	S56Y	c.167C>A	79,4	Tier-2	BM	ID	unassigned		46,XY[20]	
225	68	M	recur	K746E	c.2236A>G	54	Tier-2	BM	ID	non_preHM	*TET2,U2AF1,ZRSR2*	46,XY[20]	
226	78	M	recur	D585E	c.1755C>A	78,4	Tier-2	BM	ID	MDS	*SRSF2,TET2*	46,XY[20]	
227	66	M	recur	K746I	c.2237A>T	4,59	Tier-2	BM	ID	non_preHM	*DNMT3A,RAD21*	45,X,-Y[16]/46,XY[6]	
228	76	M	recur	Y618C	c.1853A>G	6,9	Tier-2	PB, BM	ID	MDS	*ASXL1,CBL,GNB1,MPL,SRSF2,TET2*	46,XY,t(7;8)(q32;q13)[3]/46,XY[17]	
229	89	M	recur	I301V	c.901A>G	6	Tier-2	BM	ID	MDS	*TET2*	45,X,-Y[12]/46,XY[8]	
230	61	M	recur	N139H	c.415A>C	47,5	Tier-3	PB	ID	unassigned	*CUX1,SRSF2*		
231	65	M	recur	P745_K746delinsQ	c.2234_2236del	3,27	Tier-2	BM	ID	MDS/MPN	*U2AF1*	46,XY[20]	
232	44	M	recur	N606I:I894S	c.1817A>T:c.2681T>G	7.2:64.9	Tier-2, Tier-2	BM	ID	MDS		46,XY[20]	hyper-Bil
233	79	M	nonrecur	nonM41SS	c.1575+2T>C	2,99	Tier-2	BM	ID	unassigned	*PHF6,TET2*	45,X,-Y[20]	
234	77	F	nonrecur	L259R	c.776T>G	34	Tier-3	BM	FU	MDS	*SF3B1*	46,XX[20]	
235	78	M	nonrecur	R747H	c.2240G>A	7,6	Tier-2	BM	FU	Lymphoid			
236	79	M	nonrecur	C413F	c.1238G>T	11,5	Tier-2	BM	ID	Lymphoid	*MYD88,PPM1D,TP53*	46,XY,del(5)(q14q34)[24]/46,XY[6]	
237	83	M	nonrecur	nonM41SS	c.2940+1G>T	16,7	Tier-2	PB	ID	unassigned	*DNMT3A*		
238	84	M	nonrecur	I184dup	c.551_553dup	37,1	Tier-3	PB	ID	non_preHM	*CUX1,DNMT3A,NF1,SRSF2,TET2*		
239	74	M	nonrecur	V85M	c.253G>A	4,39	Tier-2	PB	FU	AML	*ASXL1,DDX41*	no metaphases	
240	64	M	nonrecur	F926I	c.2776T>A	3,56	Tier-2	BM	ID	MDS	*CBL,SF3B1*	46,XY,del(13)(q13q21)[18]/46,XY[5]	
241	74	M	nonrecur	R69W	c.205C>T	35,4	Tier-2	BM	FU	AML	*CSF3R,NRAS*	45,X,-Y,t(8;21)(q22;q22)[20]/46,XY[1]	
242	80	M	nonrecur	M223V	c.667A>G	70,3	Tier-2	BM	ID	unassigned	*ASXL1,JAK2,U2AF1*	46,XY,der(9)dup(9)(q21q33)t(9;9)(q34;p13)[17]/46,XY[3]	
243	55	F	nonrecur	L885Q	c.2654T>A	3,28	Tier-2	PB	FU	non_preHM			
244	68	M	nonrecur	V805I	c.2413G>A	2,97	Tier-3	BM	FU	Lymphoid		46,XY[20]	
245	59	F	nonrecur	E430K	c.1288G>A	3,72	Tier-2	BM	ID	non_preHM		46,XX[21]	
246	66	F	nonrecur	A308V	c.923C>T	33,7	Tier-3	BM	FU	MDS/MPN	*CALR,TET2*	46,XX[20]	
247	80	M	nonrecur	L70H	c.209T>A	94,6	Tier-3	BM	FU	unassigned	*SRSF2*		
248	61	M	nonrecur	A887E	c.2660_2661delinsAG	62,5	Tier-2	BM	FU	unassigned	*SRSF2*		
249	78	M	nonrecur	D563del	c.1686_1688del	3	Tier-2	PB	ID	unassigned	*ASXL1*		
250	53	F	nonrecur	K68R	c.203A>G	26,2	Tier-3	BM	ID	unassigned	*DNMT3A*	46,XX[20]	
251	84	M	nonrecur	A488V	c.1463C>T	76,9	Tier-2	PB	FU	non_preHM			
252	82	F	nonrecur	D555G	c.1664A>G	4,34	Tier-3	BM	ID	AML	*BCOR,BCORL1,DNMT3A,IDH1,PHF6,RUNX1*	46,XX[15]	
253	27	M	nonrecur	E1043K	c.3127G>A	41,3	Tier-3	BM	ID	non_preHM		47,XXYc or 47,XY,+X[20]	
254	52	M	nonrecur	D623Y	c.1867G>T	15,1	Tier-2	BM	ID	unassigned	*BCOR*	46,XY,der(11)t(11;12)(q14;q24),der(12)t(12;20)(q14;p11),der(20)t(11;20)(?q22;p11)[2]/46,XY[19]	
255	70	F	nonrecur	Y425N	c.1273T>A	33,7	Tier-2	BM	FU	MPN	*TET2*	46,XX,t(9;22)(q34;q11)[7]/46,XX[15]	
256	84	F	nonrecur	G230S	c.688G>A	56,8	Tier-3	BM	ID	AML	*NF1,TP53*	43~44,XX,der(1)ins(1;7)(q21;p22p15)t(1;17)(q31;q25),der(1;3)(p36;p11),der(2)t(2;4)(q24;q27)t(3;4)(p11;q31),der(3)t(2;3)(q3	

ID	Age	Sex	Class	Variant	DNA	VAF	Tier	Mat	IDFU	Diagnosis	Comutation	Karyotype	comment
												4;p11),del(4)(q12q31),del(5)(q14q33),-7,r(7)(p11q21),der(8)t(1;8)(p11;q24),der(11)(11pter->11p15::1q31->1q32::17q12->17q25::11p15->11qter),-17 cp[14]/46,XX[10]	
257	65	M	nonrecur	V571M	c.1711G>A	5,6	Tier-2	BM	ID	unassigned	*ASXL1,CSF3R,MYD88,NRAS,U2AF1,U2AF2*	no metaphases	
258	74	M	nonrecur	V536M	c.1606G>A	28,6	Tier-2	BM	ID	non_preHM	*BRAF,NF1*	46,XY[4]	
259	83	F	nonrecur	A870Qfs*16	c.2608del	26,7	Tier-2	BM	FU	MDS	*TP53*	46,XX,del(5)(q14q34)[14]/46,XX[6]	
260	72	M	nonrecur	E129K	c.385G>A	4,45	Tier-3	BM	ID	unassigned	*PRPF8,STAG2*	45,X,-Y[6]/46,XY[15]	
261	84	M	nonrecur	A311G	c.932C>G	75	Tier-2	BM	ID	unassigned	*PPM1D,TP53*	44,X,-Y,del(5)(q13q34),del(6)(p21p11),der(7;18)(p10;q10)del(18)(q11q23),der(11)(11pter->11q25::5q13->5q14::11q22->11q23::hsr::11q23->11q23::5q33->5q34::11q22->11q23::hsr::11q23->11qter),r(13)(p11q13),der(17)t(5;17)(q34;p12),der(21)(21pter>21q22::hsr::21q22->21q22::13q13->13qter)[18]/46,XY[2]	
262	71	M	nonrecur	T832I	c.2495C>T	86,2	Tier-2	BM	ID	AML	*EZH2,FLT3.ITD,IDH2,NPM1,WT1*	46,XY[20]	
263	68	F	nonrecur	N351S	c.1052A>G	11,5	Tier-3	BM	ID	MDS	*DNMT3A*	46,XX[20]	
264	79	M	nonrecur	G84C	c.250G>T	51,2	Tier-3	BM	ID	MDS	*DNMT3A*	46,XY[20]	
265	73	M	nonrecur	*1059Cfs*58	c.3177del	14,5	Tier-2	BM	FU	non_preHM	*SRSF2,TET2*	46,XY[20]	hemolysis-sus
266	87	M	nonrecur	L997P	c.2990T>C	1,2	Tier-2	BM	ID	non_preHM	*PPM1D,RAD21,STAG2,TET2*	46,XY[9]	
267	80	M	nonrecur	Q946L	c.2837A>T	68,2	Tier-2	PB	ID	unassigned			vexas-sus
268	33	F	nonrecur	T753A	c.2257A>G	3,86	Tier-2	PB	ID	non_preHM	*ASXL1,DNMT3A,RUNX1*	no metaphases[0]	
269	71	F	nonrecur	E496K	c.1486G>A	68,6	Tier-3	BM	FU	AML	*TP53*	48,XX,t(4;8)(q28;q21),der(5)t(5;17)(q14;q11),17,del(18)(q11q23),+ider(21)(q10)del(21)(q22q22)x2,+22[7]/43,XX,der(2;16)(p15;p12),der(4;14)(8qter->8q21::4q35->4q10::14q10>14qter),der(5)t(5;17)(q14;q11),der(8)t(4;8)(q28;q21),der(11)t(2;11)(p15;q25),17,del(18)(q11q23),der(22)t(4;22)(q11;p11)t(4;8)(q35;q21)[2]/46,XX[13]	
270	66	M	nonrecur	E597V	c.1790A>T	49,7	Tier-2	BM	ID	unassigned	*GNB1*		vexas-sus
271	74	F	nonrecur	N728S	c.2183A>G	22,9	Tier-3	BM	FU	unassigned	*TET2*	46,XX[20]	
272	68	M	nonrecur	M584V	c.1750A>G	4,37	Tier-2	PB	ID	unassigned	*DDX41,PPM1D,SF3B1,TP53*		
273	76	M	nonrecur	L156del	c.465_467del	3,79	Tier-2	BM	ID	MDS	*CBL,IDH2,SF3B1*	45,X,-Y[16]/46,XY[4]	
274	59	M	nonrecur	Q665E	c.1993C>G	9,8	Tier-3	PB	ID	unassigned	*RUNX1*		
275	63	M	nonrecur	N928D	c.2782A>G	13	Tier-2	PB	FU	unassigned	*DNMT3A,SMC3,TET2*		
276	34	F	nonrecur	H713Y	c.2137C>T	3,82	Tier-3	BM	ID	non_preHM		46,XX[20]	

recur: recurrent non-M41 variants. nonrecur: Non-recurrent non-M41 variants. BM: Bone marrow. PB: Peripheral blood. ID: Initial diagnosis. FU: Follow-up. . AML: Acute myeloid leukemia. MDS: Myelodysplastic neoplasms. MDS/MPN: Myelodysplastic/myeloproliferative neoplasms. MPN: Myeloproliferative neoplasms. Lymphoid: Non-myeloid malignancies except multiple myeloma. MM: multiple myeloma. non/pre-HM: premalignant or non-diagnostic cases. unassigned: Patients with molecular genetic examination only. AML-Prog: AML progression observed in follow-up samples. CMML-Prog: CMML progression observed in follow-up samples. vexas-sus: VEXAS suspected in the referral letter. hemolysis: Documented hemolysis. hyper-Bil: hyperbilirubinemia. hemolysis-sus: hemolysis suspected in the referral letter without test results attached.
